# Maternal body mass index in early pregnancy and autism in offspring: a population-based cohort study in Sweden and Denmark

**DOI:** 10.1186/s12916-025-04487-z

**Published:** 2025-11-07

**Authors:** Matilda Morin, Weiyao Yin, Heidi MacLean, Bernie Devlin, Abraham Reichenberg, Shanna H. Swan, Joseph D. Buxbaum, Diana Schendel, Martina Persson, Thomas Munk Laursen, Alexander Kolevzon, Jakob Grove, Lambertus Klei, Kathryn Roeder, Sven Sandin

**Affiliations:** 1 Department of Medical Epidemiology and Biostatistics, Karolinska Institutet, Box 281, Stockholm, 171 77 Sweden; 2 Department of Biomedicine, Aarhus University, Aarhus, Denmark; 3 Department of Psychiatry, University of Pittsburgh School of Medicine, Pittsburgh, USA; 4 Department of Psychiatry, Icahn School of Medicine at Mount Sinai, New York, USA; 5 Seaver Autism Center for Research and Treatment, Icahn School of Medicine at Mount Sinai, New York, USA; 6 Department of Environmental Medicine and Climate Science, Icahn School of Medicine at Mount Sinai, New York, USA; 7 National Center for Registry-Based Research, Aarhus University, Aarhus, Denmark; 8 AJ Drexel Autism Institute, Drexel University, Philadelphia, USA; 9 The Lundbeck Foundation Initiative for Integrative Psychiatric Research, iPSYCH, Aarhus, Denmark; 10 Division of Pediatrics, Department of Clinical Science and Education, Karolinska Institutet, Stockholm, Sweden; 11 Bioinformatics Research Centre, BiRC, Aarhus University, Aarhus, Denmark; 12 Center for Genomics and Personalized Medicine, Aarhus, Denmark; 13 Department of Statistics and Data Science, Carnegie Mellon University, Pittsburgh, PA USA

**Keywords:** Autism spectrum disorder, Body mass index, Pregnancy, Cohort study, Registries

## Abstract

**Background:**

Elevated maternal pre-pregnancy body mass index (BMI) has been suggested to increase risk of offspring autism spectrum disorder (ASD) but evidence is mixed across heterogeneous studies and robust estimates spanning the full BMI range are lacking. This study examined the association between maternal BMI and offspring ASD in a harmonized, two-nation study and across the full BMI range.

**Methods:**

We included all singleton children born in Denmark 2004–2018 and Sweden 1998–2019 to parents of Nordic origin (*n* = 2,072,445), with follow-up from age 2 until 31 December 2021, or 2022, respectively. Maternal BMI recorded at the first antenatal visit was obtained from the Swedish and Danish Medical Birth Registers and was analyzed as a continuous variable and in World Health Organization-defined categories of underweight (BMI < 18.5), normal weight (18.5–24.9), overweight (25–29.9), obese class I (30–34.9), and obese class II–III (≥ 35). The relative risk of ASD was estimated as hazard ratios (HR) from Cox regression models, adjusted for birth year and parental age, educational level, income, and psychiatric history at time of childbirth, using data from national health and population registers. Both country-specific and pooled analyses were conducted. Subgroup and sensitivity analyses, including a sibling comparison, were performed to address the specificity and robustness of findings.

**Results:**

A total of 58,416 (2.8%) children were diagnosed with ASD during follow-up. The risk of ASD exhibited a J-shaped association with BMI, which gradually increased for mothers with both lower and higher BMI compared to BMI 22 (mid-normal range) (HR = 1.16 [95% CI 1.06–1.27] for BMI 15, and HR = 1.50 [95% CI 1.46–1.53] for BMI 30 in the fully adjusted model). Adjustment for familial factors in a sibling comparison attenuated associations.

**Conclusions:**

Both high and low maternal BMI are associated with an increased risk of ASD in the offspring. Familial factors, including genetic and environmental components consistent between siblings, may explain part of the association.

**Supplementary Information:**

The online version contains supplementary material available at 10.1186/s12916-025-04487-z.

## Background

Autism spectrum disorder (ASD) is a neurodevelopmental disorder characterized by social communication deficits and restricted, repetitive behaviors [1]. The prevalence of reported ASD diagnoses is increasing worldwide and exceeds 2% in children in the USA [2]. While both genetic and environmental factors are known to contribute to liability, the etiology of ASD is still incompletely understood [3–5].

Elevated maternal body mass index (BMI) has been suggested to increase ASD risk. Meta-analyses show positive associations between maternal pre-pregnancy overweight/obesity and offspring ASD, with a possible dose–response relationship [6, 7]. Although meta-analyses are regarded as the gold standard for synthesizing epidemiologic evidence, they necessarily integrate results from studies using different designs and analytic approaches, and the level of evidence in meta-analysis for the association between maternal obesity and ASD has been graded as weak due to high heterogeneity of existing studies [4]. Moreover, most previous studies have examined BMI in categories, often restricted to overweight and obesity, rather than analyzing the full BMI range as a continuous variable to capture the complete shape of the association [8, 9].

Several mechanisms have been proposed to explain an association between maternal BMI and ASD. Pre-clinical findings suggest that obesity-induced systemic inflammation may impair fetal neurodevelopment [10, 11]. Obesity-related pregnancy complications such as gestational diabetes [12], pre-eclampsia [13], and preterm birth [14] have also been associated with ASD [4, 15]. However, other research indicates that part of this association may be attributable to confounding, particularly through shared familial factors [8, 9].

To address key limitations in prior research, we used national health and population registers from Sweden and Denmark to construct two population-based cohorts that, together, represent the largest and most contemporary cohorts studied to date in the context of maternal BMI and ASD. These cohorts offered minimal loss to follow-up, extensive data on potential confounders, and enabled analyses with substantial statistical power and low risk of selection bias. We applied harmonized analytic strategies across both cohorts to reduce methodological heterogeneity and improve comparability, and maternal BMI was modeled across its full range. We studied the specificity of the association by sex and for ASD with co-occurring intellectual disability (ID) and attention deficit hyperactivity disorder (ADHD). We also used a sibling comparison design to assess the potential influence of unmeasured familial factors on the risk of ASD.

## Methods

### Data sources

We conducted a prospective cohort study using nationwide health registers in Sweden and Denmark. The Medical Birth Registers (MBR) in each country cover nearly all births and include data on antenatal care, delivery, and fetal outcomes [16, 17]. The National Patient Registers (NPR) contain individual-level data on medical diagnoses from inpatient and outpatient specialist care [18, 19]. The Swedish Total Population Register and the Danish Civil Registration System include data on deaths, migrations, and family relationships [20–22]. The Swedish Longitudinal Database for Health Insurance and Labour Market Studies and the Danish Integrated Database for Labour Market Research provide data on highest completed educational level and disposable income [23, 24]. Linkage between registers is possible through personal identification numbers assigned to all Swedish and Danish residents [22, 25].

### Study population

We included all live singleton births in Sweden 1998–2019 and Denmark 2004–2018 (Danish maternal weight records begin in 2004). To reduce potential genetic confounding and effect of changing immigration patterns, only individuals with parents born in the Nordic countries (Sweden, Denmark, Norway, Finland, Iceland) were included.

### Exposure

The primary exposure was maternal BMI (in kg/m^2^) in early pregnancy, obtained from the MBRs. In Sweden, BMI was calculated from measured weight at the first antenatal visit and self‑reported height; in Denmark, from self‑reported pre‑pregnancy weight and self‑reported height. In both countries, these data were recorded at the first antenatal visit, typically at 6–10 weeks’ gestation [17, 26]. BMI was analyzed both as a continuous variable and according to the World Health Organization categories: underweight (BMI < 18.5), normal weight (18.5–24.9), overweight (25.0–29.9), obese class I (30–34.9), and obese class II–III (≥ 35) [27].

### Outcome

Starting follow-up at age 2, the primary outcome was the first clinical diagnosis of ASD registered in the NPR, identified using International Classification of Diseases 10th revision codes (Additional file 1: Table S1). Diagnoses of ASD have high validity in both Sweden and Denmark [28, 29], but to further increase specificity, follow-up for ASD diagnoses started from age 2 years.

### Other variables

Potential confounders at delivery, chosen a priori based on reported ASD associations, included child birth year (in 3-year intervals), maternal and paternal age, maternal and paternal highest education (compulsory/upper secondary/university), maternal and paternal income (in quartiles), and maternal and paternal history of any diagnosis of psychiatric disease. From the MBR, we obtained child’s sex assigned at birth and gestational age. ASD with co-occurring ID or ADHD were defined as a first registered diagnosis of ID or ADHD, respectively, in the NPR within 30 days of the index ASD diagnosis (details in Additional file 1: Table S1).

### Statistical analysis

Children were followed from age 2 years until their first ASD diagnosis, emigration, death, or 31 December 2021 (Denmark) or 31 December 2022 (Sweden), whichever came first. We estimated incidence rates of ASD per 100,000 person-years and used inverse Kaplan–Meier curves to plot the cumulative incidence of ASD by maternal BMI category with maximum follow-up until age 24 in Sweden and age 17 in Denmark. In the primary analysis, we estimated the relative risk of ASD using hazard ratios from Cox proportional hazards models with robust standard errors to account for correlation between offspring from the same mother [30]. BMI was modeled using restricted cubic splines with knots at the 5th, 27.5th, 50th, 72.5th, and 95th percentiles to account for non-linearity [31]. The median BMI value in the normal weight category (i.e., 22) was used as the reference. In model 1, we adjusted for birth year only. In model 2, we further adjusted for maternal and paternal age (with restricted cubic splines), educational level, and income. In model 3, we further adjusted for maternal and paternal psychiatric history. We also modeled BMI in categories with normal weight as reference. Subjects with missing data on BMI or other covariates were excluded, i.e., a complete-case approach.

The assumption of proportional hazards for the primary exposure of interest, BMI, in the Cox model was examined by visual inspection of weighted Schoenfeld residuals [32]. All tests of statistical hypotheses were measured with a 2-sided 5% significance level. Statistical analyses were performed in SAS software v9.4.

We applied the same protocol to the Danish and Swedish data, but data sharing restrictions prohibited us from combining data into a single data set. Thus, statistical analyses were performed separately at each site by sharing the programming code, and results were subsequently pooled [33]. For both continuous and categorical BMI exposures, pooling was done using a common-effect model. Pooled effect estimates were calculated as a weighted average of the site-specific effect estimates, with weights assigned using the inverse of the variance from each site-specific estimate [33, 34].

Statistical analyses followed a pre-specified analysis plan. Pooling of results was added after the analysis had started to simplify presentation of results and to increase study power, especially for stratified analyses. However, we visually compared site-specific results at each analysis stage. The absence of marked differences supported confidence in the pooled results. All analyses and reporting adhered to the STROBE (Strengthening the Reporting of Observational Studies in Epidemiology) guidelines for observational studies (Additional file 2).

#### Supplementary analyses

Because of the sex difference in ASD risk [2], we repeated the primary analysis by offspring sex. To formally assess potential effect modification by sex, we included an interaction term between maternal BMI category and offspring sex in the unstratified regression model.

To examine the specificity of the association between maternal BMI and ASD, we compared the risk of ASD with and without co-occurring ID in Cox models where ASD with and without ID were analyzed jointly [35]. We repeated the same analysis for ASD with and without co-occurring ADHD.

To examine potential familial confounding, i.e., confounding by time-invariant factors common to all offspring of the same parents, we restricted the sample to full siblings and conducted a sibling comparison by fitting stratified Cox regression models with separate strata for each family. This model assesses the association between maternal BMI and offspring ASD within each family, removing confounding from unmeasured familial factors that are shared and stable across siblings. Such factors may include genetic predisposition, early-life environment, and aspects of socioeconomic status [36, 37]. Analyses were conducted for both continuous and categorical BMI. To assess generalizability of findings (and possible selection bias) based on the sibling sample compared to the total study population, we repeated the standard Cox regression in the sibling population, without stratification by family.

In the Swedish sibling sample, we also examined how a change in BMI between pregnancies in the same mother affected ASD risk according to the mother’s BMI category. We classified each mother by her median early-pregnancy BMI across pregnancies, from underweight to obese class II–III. For each of the woman’s pregnancies, we calculated the BMI difference relative to her median BMI. We then fitted a logistic regression model to predict the ASD risk associated with lower or higher BMI within each median BMI category.

Preterm birth is a risk factor for ASD [15] and is more common among women with overweight and obesity during pregnancy [14]. As an exploratory post hoc analysis to examine the modifying role of gestational age on the association between maternal BMI and offspring ASD, we repeated the primary analysis in subgroups by gestational age: < 35, 35–36, 37–38, 39–41, and > 41 weeks (in the Swedish cohort only). We also formally tested for effect modification by including an interaction term between maternal BMI category and gestational age in the full model. Next, we performed a mediation analysis by approximating our Cox models with logistic regression and fitting natural effects models [38] with BMI ≥ 30 as the exposure, gestational age < 39 weeks (preterm or early term) as the mediator, and any ASD diagnosis during follow-up as the outcome.

#### Sensitivity analyses

To address the differing length of follow-up in Sweden and Denmark, we repeated the primary analysis in Sweden restricting to the same birth years (2004–2018) and end of follow-up (31 December 2021) as in the Danish sample.

To address the impact of missing data, we repeated the primary analysis with BMI in categories after multiple imputation of missing exposure and covariate values [39] (in the Swedish cohort only). Twenty-five imputed datasets were created using fully conditional specification, and parameter estimates were pooled using Rubin’s rules (SAS proc mi and proc mianalyze). The imputation model included all variables from the fully adjusted Cox model, with the time-to-event variable transformed into the Nelson-Aalen estimator of cumulative hazard [40]. Continuous variables were imputed with restricted mean matching.

## Results

### Study population

We identified 1,596,120 singleton children born in Sweden 1998–2019 and 695,716 children born in Denmark 2004–2018, all with Nordic-born parents. Children who died or emigrated before age 2 (start of follow-up), and those with missing maternal BMI or covariate data, were excluded (Additional file 1: Fig. S1). The final cohort included 2,072,445 children with 21,942,619 person-years of follow-up during which 58,416 (2.8%) were diagnosed with ASD. Tables 1 and 2 show cohort characteristics by maternal BMI category in Sweden and Denmark, respectively.

**Table 1:** Swedish cohort characteristics by maternal BMI category^a^

Characteristic	Maternal BMI category in early pregnancy				
	**Underweight**	**Normal weight**	**Overweight**	**Obese class I**	**Obese class II–III**
Number of children (% of total)	28,872 (2.0)	872,699 (60.9)	359,149 (25.1)	119,567 (8.3)	52,312 (3.7)
Male sex	14,521 (50.3)	449,380 (51.5)	184,697 (51.4)	61,767 (51.7)	26,988 (51.6)
Birth year					
1998–2001	4747 (16.4)	142,643 (16.3)	56,472 (15.7)	16,373 (13.7)	6019 (11.5)
2002–2004	3538 (12.3)	118,165 (13.5)	47,606 (13.3)	14,639 (12.2)	6197 (11.8)
2005–2007	3778 (13.1)	121,669 (13.9)	49,152 (13.7)	15,696 (13.1)	6973 (13.3)
2008–2010	4161 (14.4)	130,366 (14.9)	52,472 (14.6)	17,419 (14.6)	7782 (14.9)
2011–2013	4265 (14.8)	125,317 (14.4)	51,697 (14.4)	17,889 (15.0)	7759 (14.8)
2014–2016	4447 (15.4)	122,971 (14.1)	50,650 (14.1)	18,344 (15.3)	8295 (15.9)
2017–2019	3936 (13.6)	111,568 (12.8)	51,100 (14.2)	19,207 (16.1)	9287 (17.8)
Maternal age (years)					
< 25	6789 (23.5)	101,881 (11.7)	43,794 (12.2)	17,156 (14.3)	7656 (14.6)
25–34	18,466 (64.0)	600,880 (68.9)	237,839 (66.2)	77,318 (64.7)	33,856 (64.7)
≥ 35	3617 (12.5)	169,938 (19.5)	77,516 (21.6)	25,093 (21.0)	10,800 (20.6)
Paternal age (years)					
< 25	3698 (12.8)	52,083 (6.0)	21,880 (6.1)	8251 (6.9)	3480 (6.7)
25–34	17,563 (60.8)	528,054 (60.5)	210,868 (58.7)	68,744 (57.5)	29,261 (55.9)
35–42	6505 (22.5)	251,434 (28.8)	106,420 (29.6)	35,374 (29.6)	15,938 (30.5)
≥ 43	1106 (3.8)	41,128 (4.7)	19,981 (5.6)	7198 (6.0)	3633 (6.9)
Maternal psychiatric history	4841 (16.8)	96,479 (11.1)	43,486 (12.1)	17,423 (14.6)	8710 (16.7)
Paternal psychiatric history	3026 (10.5)	61,949 (7.1)	29,485 (8.2)	12,442 (10.4)	6542 (12.5)
Maternal education					
Compulsory	3843 (13.3)	54,360 (6.2)	27,504 (7.7)	12,198 (10.2)	6473 (12.4)
Upper secondary	12,117 (42.0)	336,051 (38.5)	169,195 (47.1)	64,726 (54.1)	30,466 (58.2)
University	12,912 (44.7)	482,288 (55.3)	162,450 (45.2)	42,643 (35.7)	15,373 (29.4)
Paternal education					
Compulsory	3729 (12.9)	72,810 (8.3)	36,656 (10.2)	15,263 (12.8)	7579 (14.5)
Upper secondary	14,015 (48.5)	415,145 (47.6)	204,388 (56.9)	75,126 (62.8)	34,215 (65.4)
University	11,128 (38.5)	384,744 (44.1)	118,105 (32.9)	29,178 (24.4)	10,518 (20.1)
Maternal disposable income^b^					
Q1	10,740 (37.2)	201,789 (23.1)	88,938 (24.8)	35,202 (29.4)	17,982 (34.4)
Q2	6801 (23.6)	207,635 (23.8)	96,450 (26.9)	35,376 (29.6)	15,835 (30.3)
Q3	5530 (19.2)	220,549 (25.3)	94,325 (26.3)	29,242 (24.5)	11,848 (22.6)
Q4	5801 (20.1)	242,726 (27.8)	79,436 (22.1)	19,747 (16.5)	6647 (12.7)
Paternal disposable income^b^					
Q1	9511 (32.9)	204,940 (23.5)	88,515 (24.6)	33,900 (28.4)	17,218 (32.9)
Q2	6443 (22.3)	205,212 (23.5)	98,715 (27.5)	36,012 (30.1)	16,092 (30.8)
Q3	6075 (21.0)	216,680 (24.8)	95,186 (26.5)	30,769 (25.7)	12,714 (24.3)
Q4	6843 (23.7)	245,867 (28.2)	76,733 (21.4)	18,886 (15.8)	6288 (12.0)

*Abbreviations*: *BMI *Body mass index, *Q1 to Q4 *Quartiles

^a^Data are expressed as number (percentage) unless otherwise indicated. BMI categories were defined according to the World Health Organization as BMI < 18.5, underweight; BMI 18.5–24.9, normal weight; BMI 25.0–29.9, overweight; BMI 30–34.9, obese class I; and BMI ≥ 35, obese class II–III

^b^In quartiles per income year

**Table 2:** Danish cohort characteristics by maternal BMI category^a^

Characteristic	Maternal BMI category in early pregnancy				
	**Underweight**	**Normal weight**	**Overweight**	**Obese class I**	**Obese class II–III**
Number of children (% of total)	25,060 (3.9)	394,799 (61.7)	136,111 (21.3)	54,120 (8.5)	29,756 (4.7)
Male sex	12,857 (51.3)	203,068 (51.4)	69,694 (51.2)	27,677 (51.1)	15,148 (50.9)
Birth year					
2004	1758 (7.0)	29,208 (7.4)	9499 (7.0)	3418 (6.3)	1689 (5.7)
2005–2007	5273 (21.0)	86,521 (21.9)	28,557 (21.0)	10,631 (19.6)	5637 (18.9)
2008–2010	5178 (20.7)	82,908 (21.0)	28,860 (21.2)	11,222 (20.7)	6309 (21.2)
2011–2013	4740 (18.9)	73,539 (18.6)	26,147 (19.2)	10,687 (19.7)	5824 (19.6)
2014–2016	4980 (19.9)	74,548 (18.9)	25,297 (18.6)	10,539 (19.5)	5838 (19.6)
2017–2018	3131 (12.5)	48,075 (12.2)	17,751 (13.0)	7623 (14.1)	4459 (15.0)
Maternal age (years)					
< 25	5194 (20.7)	40,542 (10.3)	16,032 (11.8)	7468 (13.8)	4298 (14.4)
25–34	16,389 (65.4)	278,574 (70.6)	94,039 (69.1)	36,677 (67.8)	20,086 (67.5)
≥ 35	3477 (13.9)	75,683 (19.2)	26,040 (19.1)	9975 (18.4)	5372 (18.1)
Paternal age (years)					
< 25	3024 (12.1)	21,385 (5.4)	8036 (5.9)	3463 (6.4)	1933 (6.5)
25–34	15,124 (60.4)	244,012 (61.8)	83,395 (61.3)	32,633 (60.3)	17,471 (58.7)
35–42	5988 (23.9)	112,520 (28.5)	38,926 (28.6)	15,606 (28.8)	8975 (30.2)
≥ 43	924 (3.7)	16,882 (4.3)	5754 (4.2)	2418 (4.5)	1377 (4.6)
Maternal psychiatric history	5183 (20.7)	53,269 (13.5)	19,361 (14.2)	8756 (16.2)	5546 (18.6)
Paternal psychiatric history	3162 (12.6)	33,868 (8.6)	13,806 (10.1)	6407 (11.8)	4332 (14.6)
Maternal education					
Compulsory	6568 (26.2)	50,570 (12.8)	22,001 (16.2)	11,564 (21.4)	7734 (26.0)
Upper secondary	8449 (33.7)	144,593 (36.6)	59,048 (43.4)	25,382 (46.9)	14,058 (47.2)
University	10,043 (40.1)	199,636 (50.6)	55,062 (40.5)	17,174 (31.7)	7964 (26.8)
Paternal education					
Compulsory	5948 (23.7)	56,429 (14.3)	25,168 (18.5)	12,909 (23.9)	8706 (29.3)
Upper secondary	10,277 (41.0)	179,669 (45.5)	73,023 (53.6)	30,335 (56.1)	16,403 (55.1)
University	8835 (35.3)	158,701 (40.2)	37,920 (27.9)	10,876 (20.1)	4647 (15.6)
Maternal disposable income^b^					
Q1	8757 (34.9)	90,155 (22.8)	31,668 (23.3)	14,438 (26.7)	9219 (31.0)
Q2	5902 (23.6)	90,107 (22.8)	38,290 (28.1)	17,331 (32.0)	10,036 (33.7)
Q3	4873 (19.4)	101,032 (25.6)	36,432 (26.8)	13,499 (24.9)	6758 (22.7)
Q4	5528 (22.1)	113,505 (28.8)	29,721 (21.8)	8852 (16.4)	3743 (12.6)
Paternal disposable income^b^					
Q1	7979 (31.8)	91,429 (23.2)	31,925 (23.5)	14,058 (26.0)	8660 (29.1)
Q2	5284 (21.1)	90,825 (23.0)	39,172 (28.8)	17,091 (31.6)	10,016 (33.7)
Q3	5300 (21.1)	100,048 (25.3)	36,088 (26.5)	13,860 (25.6)	7109 (23.9)
Q4	6497 (25.9)	112,497 (28.5)	28,926 (21.3)	9111 (16.8)	3971 (13.3)

*Abbreviations*: *BMI* Body mass index, *Q1 to Q4* Quartiles

^a^Data are expressed as number (percentage) unless otherwise indicated. BMI categories were defined according to the World Health Organization as BMI < 18.5, underweight; BMI 18.5–24.9, normal weight; BMI 25.0–29.9, overweight; BMI 30–34.9, obese class I; and BMI ≥ 35, obese class II–III

^b^In quartiles per income year

### Maternal BMI and risk of ASD

**Fig. 1:**
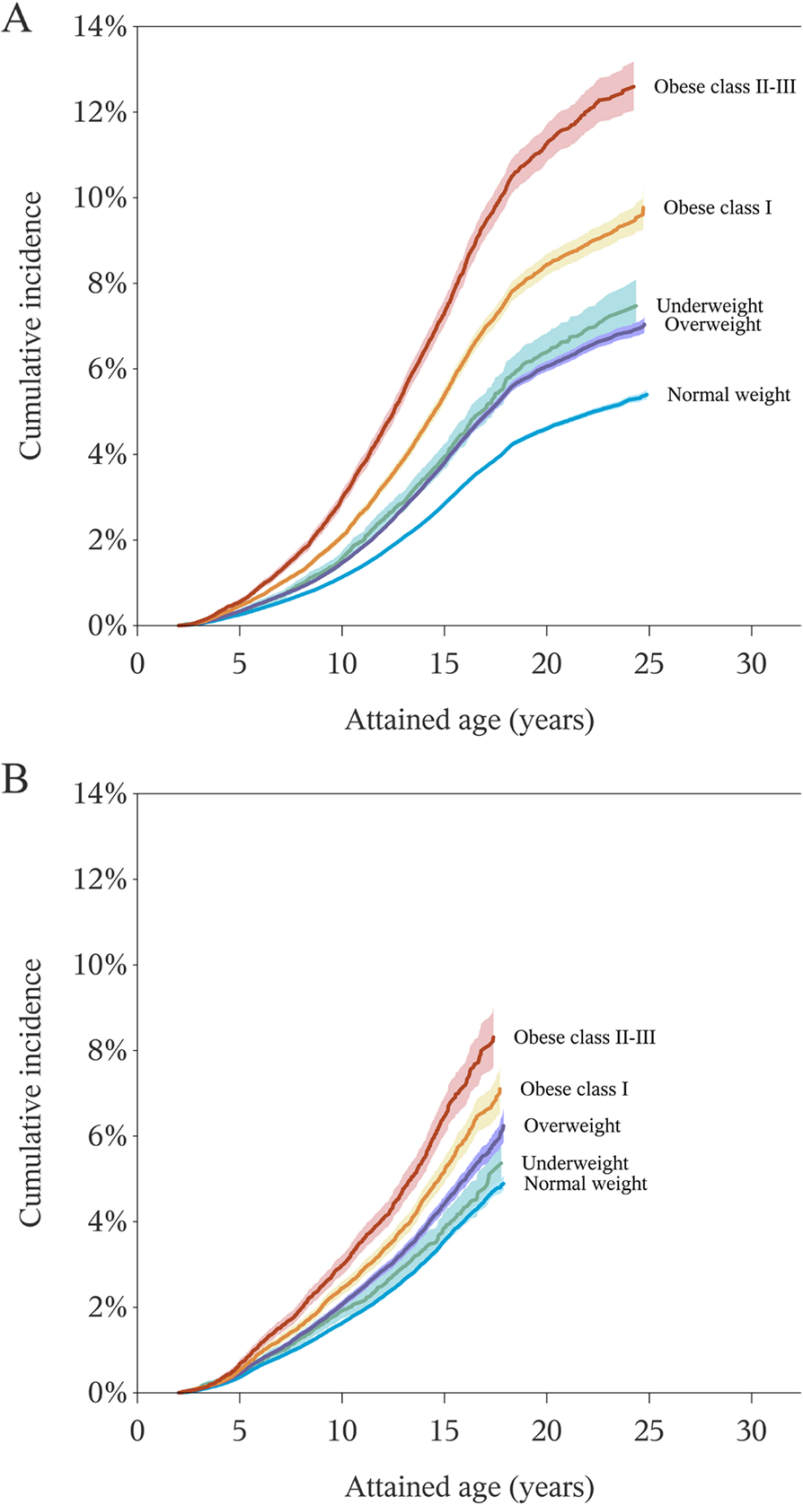
Inversed Kaplan–Meier curves for age-cumulative incidence of autism spectrum disorder by maternal body mass index category in **A** Sweden and **B** Denmark. Presented by site due to data pooling restrictions. Shaded bands indicate pointwise two-sided 95% confidence intervals. BMI categories were defined according to the World Health Organization as BMI < 18.5, underweight; BMI 18.5–24.9, normal weight; BMI 25.0–29.9, overweight; BMI 30–34.9, obese class I; and BMI ≥ 35, obese class II–III. Maximum follow-up until age 24 in Sweden and age 17 in Denmark due to differences in data availability

The crude incidence rate of ASD diagnosis across sites was 266.2 per 100,000 person-years. As reflected in the inverse Kaplan–Meier curves (Fig. 1), the cumulative incidence of ASD by age was higher for overweight compared to normal weight, higher still for obese class I and highest for obese class II–III. The cumulative ASD incidence was also higher among offspring of underweight compared to normal weight mothers. Across all BMI categories, the separation between curves for each BMI category appeared more pronounced in Sweden than in Denmark.

**Fig. 2:**
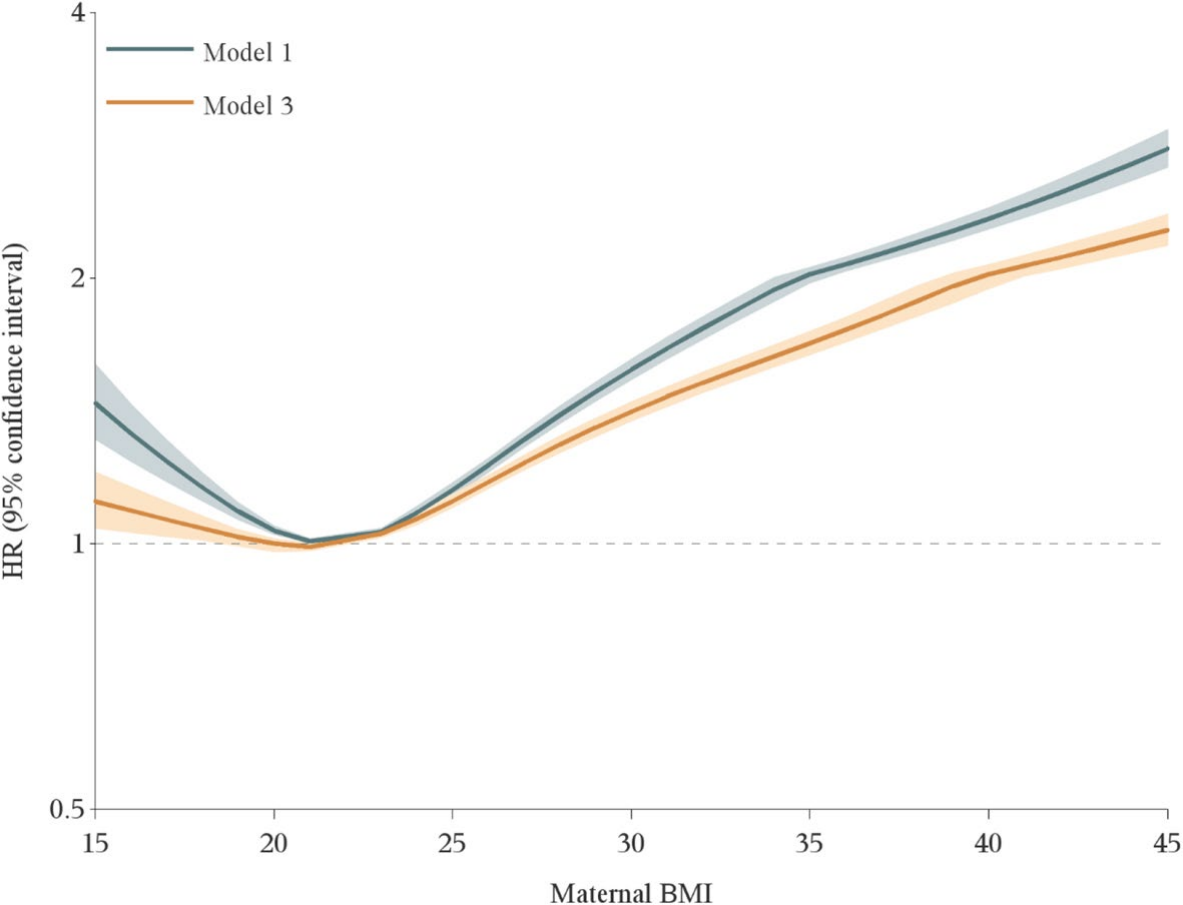
Maternal body mass index (BMI) in early pregnancy and pooled relative risk of offspring autism spectrum disorder. Hazard ratios (HRs) from Cox regression models with attainted age of child as underlying time scale, adjusted for birth year (model 1; upper gray curve), plus parental age, educational level, disposable income, and psychiatric history (fully adjusted model, model 3: lower orange curve). BMI was modeled with a restricted cubic spline, using the median BMI value in the “normal weight” category, i.e., 22, as reference. Shaded bands indicate 95% confidence intervals

**Fig. 3:**
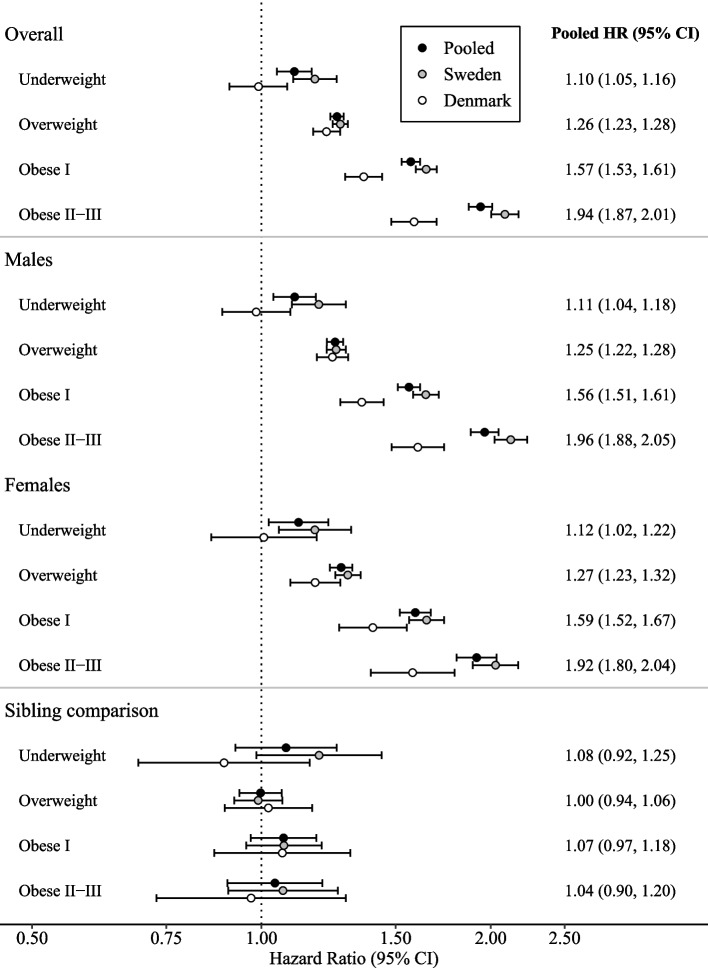
Maternal body mass index and risk of offspring autism spectrum disorder, overall and in subgroups. Dots represent hazard ratios (HRs) with 95% confidence interval bars from Cox regression models with attainted age of child as underlying time scale, adjusted for birth year, parental age, educational level, disposable income, and psychiatric history (model 3). Results from sibling comparison are from a stratified Cox regression model clustered on the mother to remove confounding from factors shared within families. BMI categories were defined according to the World Health Organization as BMI < 18.5, underweight; BMI 18.5–24.9, normal weight; BMI 25.0–29.9, overweight; BMI 30–34.9, obese class I; and BMI ≥ 35, obese class II–III

The pooled relative risk of ASD from Cox proportional hazard models with continuous BMI exhibited a J-shaped association, with the lowest risk at BMI 22 and increasing risks for lower and higher BMI values (Fig. 2). In the fully adjusted model, the HR of ASD for a BMI of 15 compared to 22 was 1.16 (95% CI, 1.06–1.27), and for a BMI of 30 the HR was 1.50 (95% CI, 1.46–1.53). When modeling BMI in categories, maternal underweight, overweight, and obesity, versus normal weight, were all associated with increased ASD risk, though associations were slightly reduced after adjusting for parental covariates (Fig. 3; Additional file 1: Table S2). There was no evidence for non-proportional hazards (Additional file 1: Figs. S2 and S3). Larger relative risks were observed for Sweden than for Denmark in most analyses. However, as raw data could not be combined across countries, we could not formally test for interaction by country. Site-specific results are summarized in Additional file 1: Supplementary results, Table S3, and Figs. S4–S7.

### Supplementary analyses

The pooled relative risk of ASD associated with maternal BMI category did not differ by offspring sex (Fig. 3). Formal tests for effect modification by sex, conducted separately within each cohort, were not statistically significant (Sweden: *p* = 0.061; Denmark: *p* = 0.357).

The association between maternal BMI category and ASD with co-occurring ID did not differ from the association for ASD without ID, although CIs were wide (Additional file 1: Fig. S3A). ASD with ADHD tended to show stronger associations with maternal overweight/obesity than ASD without ADHD (Additional file 1: Fig. S3B).

For the sibling analyses, we identified 1,435,471 siblings from 648,518 Swedish or Danish families. Overall, 227,445 sibling pairs (23.9%) were discordant for maternal BMI category. Among these, the median absolute difference in maternal BMI was 2.6 (IQR 1.6–4.2) in Sweden and 2.9 (IQR 1.7–3.6) in Denmark. The corresponding value among concordant pairs was 0.8 (IQR 0.4–1.5) and 0.7 (0.3–1.5), respectively. Restricting the Cox regression to the sibling population did not markedly change results compared to the primary analysis (Additional file 1: Table S3). However, when analyses were stratified by family to adjust for unmeasured familial factors, associations between maternal BMI and ASD were no longer statistically significant in any BMI category (Fig. 3; Additional file 1: Table S3). The stratified analysis included the entire sibling population, with 8212 ASD events within discordant risk sets contributing informative data. Treating BMI as a continuous exposure in the sibling comparison also resulted in a null association (Additional file 1: Fig. S6). In the categorical BMI analysis in Sweden, the point estimate for maternal underweight was similar in the full sibling population (HR 1.20, 95% CI 1.11–1.31) and the family‑stratified sibling analysis (HR 1.19, 95% CI 0.98–1.44), though the stratified estimate was less precise (Additional file 1: Tables S2 and S3).

For normal/overweight mothers (median BMI across pregnancies), offspring ASD risk increased slightly after a pregnancy where BMI was lower than her median (in the Swedish cohort). For obese mothers (median BMI corresponding to obese classes I–III), the risk remained stable despite lower or higher BMI values in individual pregnancies (Additional file 1: Fig. S9). Results for underweight were inconclusive due to low power.

In the post hoc analysis stratified by gestational age in the Swedish cohort, there was little support for a difference by gestational age in the association between maternal BMI and ASD (Additional file 1: Table S4). Consistent with the results from the stratified analysis, a formal test for effect modification by gestational age did not reach statistical significance (*p* = 0.812). There was only minor mediation of risk by preterm and early-term birth (Additional file 1: Table S5).

### Sensitivity analyses

Restricting the Swedish data to the same birth years as in Denmark did not markedly alter the Swedish results compared to the primary analysis (Additional file 1: Table S6).

After multiple imputation of missing BMI and covariate values in the Swedish cohort, we repeated the primary analysis with categorical BMI, and the estimated relative risk remained robust (Additional file 1: Table S7). The analysis included 1,587,654 children, of whom 155,055 (9.8%) were missing data on maternal BMI or covariates (Additional file 1: Table S8 for cohort characteristics including subjects with missing data).

## Discussion

In this prospective study using two nationwide cohorts of over 2 million children, we found an association between maternal early-pregnancy BMI and offspring ASD that increased continuously as BMI deviated from 22 (mid-normal range), for both lower and higher BMI values. The association remained robust after adjustment for potential individual-level confounders. Among siblings, however, there were no differences in ASD risk associated with maternal BMI, suggesting that factors shared by family members may explain part of the association. Our study is distinguished by the high precision of the large, pooled sample size and the replication of the analyses in two countries.

Our study confirms and extends previous population-based studies showing a moderate effect of maternal overweight/obesity on ASD risk. The continuously increasing risk, peaking at the highest BMI category, supports a dose–response relationship that has been reported in some studies [8, 41, 42], but not replicated in others [43, 44]. Meta-analyses do not support an association between maternal underweight and ASD [6, 42], though two recent studies, including one from Denmark, reported similar findings to our pooled estimates [41, 43]. In our study, the association with maternal underweight was observed in the Swedish cohort, which drove the pooled result, but not in the Danish cohort. The reason for this discrepancy is unclear. It remained after restricting the Swedish cohort to the same birth years as the Danish cohort. Another possible explanation is measurement error in the exposure as a result of maternal BMI in Denmark being self-reported. Validation studies show that underweight women on average tend to over-report pre-pregnancy weights, while women of normal weight and above under-report, which could lead to attenuation of the association [45, 46]. This type of reporting error may partly account for the lower relative risks observed in Denmark relative to Sweden across most analyses.

The mechanisms linking maternal BMI and ASD remain unclear and may differ between low and high BMI. If the attenuation observed in the sibling analyses reflects true absence of association, this would suggest that shared familial factors may underlie the associations seen in the population sample. Maternal BMI at age 18 (but not at conception) and paternal BMI have both been linked to ASD [9, 47], supporting the hypothesis that factors correlated with BMI, potentially genetic, contribute to at least some of the ASD risk associated with maternal BMI. Obesity-induced processes in the mother such as systemic inflammation, insulin resistance, and oxidative stress may also contribute to fetal neurodevelopmental risk [11, 48, 49]. The role of neuroinflammation in ASD is supported by brain imaging and findings of elevated systemic cytokine levels in individuals with ASD [50]. Maternal obesity is also linked to gestational diabetes, pre-eclampsia, and preterm birth, all potential ASD risk factors [4, 15, 51], though our results show limited support for preterm birth as an important mediator. For underweight, genetic correlation between ASD and eating disorders exist [52], but adjusting for parental psychiatric history had little impact. Low maternal weight may also reflect other diseases or nutritional deficiencies that affects fetal neurodevelopment [53]. The elevated point estimate for underweight in Sweden observed here persisted in the sibling comparison, although it was no longer statistically significant, a pattern more consistent with loss of power than clear attenuation. Spline-based continuous analyses in siblings showed no association at low BMI, but spline fits can be sensitive to knot placement and to sparse data in the tails, which could mask effects at extremes. Whether underweight and overweight reflect distinct etiologies or a shared etiologic continuum in relation to offspring ASD should be addressed in future studies.

Our finding of no association between maternal BMI and ASD within full siblings aligns with a previous (partially overlapping) Swedish study in which the association was attenuated in a sibling comparison [8], indicative of familial confounding. However, sibling designs have important limitations that warrant consideration. Not all siblings are informative in the analysis. In stratified Cox regression, events (here ASD diagnoses) in an index person contribute only if the family includes at least one exposure-discordant sibling still at risk of ASD at the same age [37]. For exposures that are highly correlated within siblings, this is a strong restriction that reduces power and increases the risk of confounding by non-shared factors in discordant pairs [54]. For example, large changes in maternal BMI (which contribute most to effect estimates) may be a result of maternal disease or stress, which could in turn influence ASD risk in ways that differ between siblings. Cross-over effects, where the outcome of one sibling affects the exposure or outcome of another sibling, can also bias estimates, but are difficult to test for because non‑shared unmeasured confounding can generate similar, or canceling, patterns [55]. Random measurement error in the exposure has a proportionally greater impact in sibling designs than in population analyses because misclassification of truly concordant siblings as discordant contributes only noise to within‑pair contrasts, diluting associations more than in a population analysis where all individuals contribute information [54]. This bias increases with increasing exposure correlation within siblings and decreasing exposure reliability and can falsely be interpreted as familial confounding [56].

Given the disproportionate impact of measurement error in sibling designs, we evaluated its impact on our findings. In this study, we observed an attenuation of effect estimates in the sibling analyses in both Sweden and Denmark. In Denmark, maternal weight is self‑reported in early pregnancy. Self‑reported weight is often slightly inaccurate, with a tendency for women with higher BMI to under‑report and those with lower BMI to over‑report [45, 46]. Of note, any such bias that is consistent across a woman’s pregnancies would be accounted for in the sibling comparison, but any pregnancy‑specific, variable component of measurement error would remain and could still bias the results. In contrast to Denmark, maternal weight in Sweden is objectively measured at the first antenatal visit, providing high reliability. Minor variation from pregnancy‑related weight gain due to differences in gestational week at measurement is unlikely to meaningfully reduce reliability in the Swedish data. Additionally, similar attenuation in both Denmark and Sweden was seen in the sibling analysis using BMI as a continuous variable, where more sibling pairs are informative for the analysis. The reliability of the Swedish BMI measures are further supported by another Swedish study using the same data sources to examine the association between maternal BMI and offspring bipolar disorder (another condition with high sibling correlation). In that study, no attenuation was observed in the sibling comparison, although reduced power rendered the association non‑significant [57]. Taken together, these findings make it unlikely that measurement error alone accounts for the attenuation seen in the sibling analyses.

Our study has multiple strengths. It includes contemporary, accurate clinical diagnoses of ASD and takes advantage of Sweden’s and Denmark’s comprehensive health and population registers, minimizing selection bias by covering almost all children born over 21 and 15 years, respectively. These registers allowed us to adjust for important confounders like parental socioeconomic status and psychiatric history. The cohort’s biological kinship data enabled a sibling comparison design that removes confounding from factors that are difficult to measure and adjust for, such as early environment and maternal characteristics. By applying harmonized analyses across two large national cohorts, we derived pooled estimates that are more interpretable and contextually coherent than those from meta-analyses of methodologically diverse studies. This approach enhances statistical precision, supports generalizability, and addresses concerns about bias and reproducibility in observational research [58].

In addition to the limitations discussed above, including self-reported pre-pregnancy BMI and constraints of the sibling analysis, other limitations should be acknowledged. Our approach focused on minimizing confounding through design and adjustment, but we did not attempt to estimate causal effects due to limitations in available data and the complexity of modeling causal pathways. Although the Swedish and Danish register data offer extensive coverage and longitudinal depth, there may still be residual confounding due to lack of data on factors such as preconceptional folate use and gestational weight gain [59]. Restricting the cohort to children with two Nordic‑born parents reduced heterogeneity in genetic background and the influence of changing immigration patterns, but excluded about 21% of the Danish and 28% of the Swedish birth cohorts (before other exclusions), which could affect the generalizability of our findings to groups with parental origins outside the Nordic countries. Furthermore, as both Sweden and Denmark are high-income countries with similar health care structures, generalizability to other settings cannot be guaranteed.

## Conclusions

Both high and low maternal BMI are associated with an increased risk of ASD in the offspring. Familial factors, including genetic and environmental components consistent between siblings, may explain part of the association.

## Supplementary Information

Additional file 1: Supplementary results, Tables S1–S8, and Figures S1–S9. Supplementary results—from site-specific replication. Table S1. Diagnosis definitions. Table S2. Relative risk of ASD by maternal BMI, overall and by site. Table S3. Relative risk of ASD in full siblings, by site. Table S4. Relative risk of ASD by gestational week (Sweden). Table S5. Mediation by preterm and early-term birth (Sweden). Table S6. Relative risk of ASD for birth years 2004–2018 (Sweden). Table S7. Relative risk of ASD from imputed data (Sweden). Table S8. Cohort characteristics including subjects with missing data (Sweden). Fig. S1. Cohort selection flow diagram. Fig. S2. Schoenfeld residuals (Sweden). Fig. S3. Schoenfeld residuals (Denmark). Fig. S4. Relative risk of ASD by continuous BMI, by site. Fig. S5. Relative risk of ASD with co-occurring ID or ADHD, by site. Fig. S6. Relative risk of ASD in full siblings by continuous BMI, by site. Fig. S7. Relative risk of ASD by offspring sex and continuous BMI, by site. Fig. S8. Pooled relative risk of ASD with co-occurring ID or ADHD. Fig. S9. Predicted ASD risk by BMI deviation from maternal median (Sweden).

Additional file 2: STROBE checklist.

## Data Availability

Data used in this study is not publicly available. Access to data from Swedish and Danish national registers is only granted after ethical review by appropriate authorities.

## Abbreviations

ADHD: Attention deficit hyperactivity disorder
ASD: Autism spectrum disorder
BMI: Body mass index
CI: Confidence interval
HR: Hazard ratio
ID: Intellectual disability
MBR: Medical Birth Register
NPR: National Patient Register
