# Supplementary material for: Maternal body mass index in early pregnancy and autism in offspring: a population-based cohort study in Sweden and Denmark
Source: BMC Med. 2025 Nov 7;23:620. doi: 10.1186/s12916-025-04487-z (PMC12593900; doi:10.1186/s12916-025-04487-z)
Supplement: Supplementary file 1 — Additional file 1: Supplementary results, Tables S1–S8, and Figures S1–S9. Supplementary results—from site-specific replication. Table S1. Diagnosis definitions. Table S2. Relative risk of ASD by maternal BMI, overall and by site. Table S3. Relative risk of ASD in full siblings, by site. Table S4. Relative risk of ASD by gestational week (Sweden). Table S5. Mediation by preterm and early-term birth (Sweden). Table S6. Relative risk of ASD for birth years 2004–2018 (Sweden). Table S7. Relative risk of ASD from imputed data (Sweden). Table S8. Cohort characteristics including subjects with missing data (Sweden). Fig. S1. Cohort selection flow diagram. Fig. S2. Schoenfeld residuals (Sweden). Fig. S3. Schoenfeld residuals (Denmark). Fig. S4. Relative risk of ASD by continuous BMI, by site. Fig. S5. Relative risk of ASD with co-occurring ID or ADHD, by site. Fig. S6. Relative risk of ASD in full siblings by continuous BMI, by site. Fig. S7. Relative risk of ASD by offspring sex and continuous BMI, by site. Fig. S8. Pooled relative risk of ASD with co-occurring ID or ADHD. Fig. S9. Predicted ASD risk by BMI deviation from maternal median (Sweden). [file 12916_2025_4487_MOESM1_ESM.docx]

**Additional file 1: Supplementary material**

**Supplement to:** Maternal Body Mass Index in Early Pregnancy and Autism in Offspring: A Population-Based Cohort Study in Sweden and Denmark. Morin M. et al.

**List of contents:**

**Supplementary results**

**Table S1** Definitions of diagnoses

**Table S2** Maternal BMI category and relative risk of offspring ASD, overall and by site

**Table S3** Maternal BMI category and relative risk of offspring ASD in full siblings by site

**Table S4** Maternal BMI category and relative risk of offspring ASD in subgroups by gestational week in Sweden

**Table S5** Mediation of the association between maternal obesity and offspring ASD by preterm and early-term birth in Sweden

**Table S6** Maternal BMI category and relative risk of offspring ASD for birth years 2004-2018 in Sweden

**Table S7** Maternal BMI category and relative risk of offspring ASD from multiply imputed data in Sweden

**Table S8** Cohort characteristics by maternal BMI category including subjects with missing data in Sweden

**Figure S1** Flow diagram over study cohort selection

**Figure S2** Scaled Schoenfeld residual plots to assess proportional hazards assumption (Swedish cohort)

**Figure S3** Scaled Schoenfeld residual plots to assess proportional hazards assumption (Danish cohort)

**Figure S4** Maternal body mass index (BMI) (continuous) and relative risk of offspring autism spectrum disorder in (A) Sweden and (B) Denmark

**Figure S5** Maternal body mass index (BMI) category and relative risk of offspring autism spectrum disorder (ASD) with co-occurring intellectual disability (ID) or attention deficit hyperactivity disorder (ADHD) in Sweden and Denmark

**Figure S6** Maternal body mass index (BMI) (continuous) and relative risk of offspring autism spectrum disorder in full siblings in (A) Sweden and (B) Denmark

**Figure S7** Maternal body mass index (BMI) (continuous) and relative risk of offspring autism spectrum disorder by offspring sex in (A) Sweden and (B) Denmark

**Figure S8** Maternal body mass index (BMI) category and pooled relative risk of offspring autism spectrum disorder (ASD) with co-occurring (A) intellectual disability (ID), or (B) attention deficit hyperactivity disorder (ADHD)

**Figure S9** Predicted probability of offspring autism spectrum disorder in relation to maternal body mass index (BMI) difference in a specific pregnancy from the mother’s median BMI over all her pregnancies, by categories of median maternal BMI (Swedish cohort)

**SUPPLEMENTARY RESULTS**

**Summary of results from site-specific replication**

The crude incidence rate of ASD diagnosis was 263 cases per 100,000 person-years in Sweden, and 276 cases per 100,000 person-years in Denmark. The crude ASD rate in offspring of normal weight mothers, the reference category, was 219 cases per 100,000 person-years in Sweden compared to 245 in Denmark (Table S2). The relative risk of ASD comparing overweight-to-obese mothers to normal weight displayed a similar shape in Sweden and Denmark, though the magnitude was lower in Denmark. In Sweden, but not Denmark, maternal underweight was also statistically significantly associated with increased ASD risk (Figure S4). Similar patterns between countries were also seen for ASD with co-occurring ID and ADHD (Figure S5) and in stratified sibling analysis (Table S3; Figure S6 for continuous BMI). The pattern of no sex differences in the association of maternal BMI with ASD risk was replicated in both countries (Figure 3; Figure S7 for continuous BMI).

**SUPPLEMENTARY TABLES:**

**Table S1** Definition of diagnoses

| **Diagnosis** | **ICD codes** | **Definition** |
| --- | --- | --- |
| **Diagnoses in children** | **ICD-10** |  |
| ASD | F84.0, F84.1, F84.5, F84.8, F84.9 | First registered diagnosis (primary or secondary) from age 2 in the NPR |
| ASD with ID | ID: F70, F71, F72, F73, F78, F79 | A registered diagnosis (primary or secondary) of ID within 30 days of first ASD diagnosis in the NPR |
| ASD with ADHD | ADHD: F90 | A registered diagnosis (primary or secondary) of ADHD within 30 days of first ASD diagnosis in the NPR |
| **Diagnoses in parents** |  |  |
| Psychiatric history | ICD-10: F10-F99  ICD-9: 291, 295, 296, 297, 298, 299, 300, 301, 302, 303, 304, 305, 306, 307, 308, 309, 311, 312, 313, 314, 315, 316, 317, 318, 319  ICD-8: 291, 295, 296, 297, 298, 299, 300, 301, 302, 303, 304, 305, 306, 307, 308, 310, 311, 312, 313, 314, 315 | A registered diagnosis in the NPR any time before childbirth |

Abbreviations: ICD, International Classification of Diseases; ASD, autism spectrum disorder; ID, intellectual disability; ADHD, attention deficit hyperactivity disorder; NPR, Swedish National Patient Register.

**Table S2** Maternal BMI category and relative risk of offspring ASD overall and by site

| **Group** | **BMI categories^a^** | **Rate (cases/ 100,000 person years)** | **ASD cases/person-years** | **Model 1^b^  HR (95% CI)** | **Model 2^c^  HR (95% CI)** | **Model 3^d^ HR (95% CI)** |
| --- | --- | --- | --- | --- | --- | --- |
| **Pooled** | Underweight | N/A | N/A | 1.29 (1.22, 1.36) | 1.13 (1.07, 1.19) | 1.10 (1.05, 1.16) |
|  | Normal weight | N/A | N/A | Reference | Reference | Reference |
|  | Overweight | N/A | N/A | 1.30 (1.28, 1.33) | 1.25 (1.23, 1.28) | 1.26 (1.23, 1.28) |
|  | Obese class I | N/A | N/A | 1.74 (1.69, 1.79) | 1.57 (1.53, 1.61) | 1.57 (1.53, 1.61) |
|  | Obese class II-III | N/A | N/A | 2.28 (2.20, 2.36) | 1.95 (1.88, 2.02) | 1.94 (1.87, 2.01) |
| **Sweden** | Underweight | 303.5 | 994/327,525 | 1.40 (1.32, 1.50) | 1.20 (1.13, 1.28) | 1.18 (1.10, 1.26) |
|  | Normal weight | 218.8 | 22,236/10,164,956 | Reference | Reference | Reference |
|  | Overweight | 288.7 | 11,847/4,104,057 | 1.32 (1.29, 1.35) | 1.27 (1.24, 1.30) | 1.27 (1.24, 1.30) |
|  | Obese class I | 399.9 | 5,192/1,298,312 | 1.84 (1.79, 1.90) | 1.65 (1.60, 1.70) | 1.65 (1.59, 1.70) |
|  | Obese class II-III | 540.9 | 2,938/543,198 | 2.49 (2.39, 2.59) | 2.10 (2.02, 2.19) | 2.09 (2.00, 2.18) |
| **Denmark** | Underweight | 270.4 | 579/214,147 | 1.11 (1.02, 1.21) | 1.02 (0.93, 1.11) | 0.99 (0.91, 1.08) |
|  | Normal weight | 245.2 | 8,419/3,433,013 | Reference | Reference | Reference |
|  | Overweight | 305.5 | 3,559/1,165,090 | 1.25 (1.20, 1.30) | 1.21 (1.16, 1.26) | 1.22 (1.17, 1.27) |
|  | Obese class I | 355.6 | 1,600/449,950 | 1.47 (1.39, 1.55) | 1.36 (1.28, 1.44) | 1.36 (1.29, 1.44) |
|  | Obese class II-III | 434.0 | 1,052/242,371 | 1.80 (1.69, 1.93) | 1.59 (1.49, 1.70) | 1.59 (1.48, 1.70) |

Abbreviations: ASD, autism spectrum disorder; BMI, body mass index; CI, confidence interval; HR, hazard ratio.

^a^ BMI categories were defined according to the World Health Organization as BMI <18.5, underweight; BMI 18.5-24.9, normal weight; BMI 25.0-29.9, overweight; BMI 30-34.9, obese class I; BMI ≥35, obese class II-III.

^b^ Cox regression model with attainted age of child as underlying time scale, adjusted for birth year.

^c^ Adjusted for birth year, parental age, educational level, and disposable income.

^d^ Adjusted for birth year, parental age, educational level, disposable income, and psychiatric history.

**Table S3** Maternal BMI category and relative risk of offspring ASD in full siblings by site

| **Country** | **BMI categories^a^** | **Sibling population ^b^  (Cox regression)** | | | **Sibling comparison ^c^ (Stratified Cox regression)** | | |
| --- | --- | --- | --- | --- | --- | --- | --- |
|  |  | **N events** | **Model 1^d^**  **HR (95% CI)** | **Model 3^e^**  **HR (95% CI)** | **N informative events^f^** | **Model 1^d^**  **HR (95% CI)** | **Model 3^e^**  **HR (95% CI)** |
| **Sweden** | Underweight | 626 | 1.40 (1.28, 1.52) | 1.20 (1.11, 1.31) | 228 | 1.15 (0.95, 1.39) | 1.19 (0.98, 1.44) |
|  | Normal weight | 14,368 | Reference | Reference | 1,779 | Reference | Reference |
|  | Overweight | 7,519 | 1.35 (1.31, 1.39) | 1.29 (1.26, 1.33) | 2,422 | 1.00 (0.93, 1.08) | 0.99 (0.92, 1.06) |
|  | Obese class I | 3,284 | 1.91 (1.84, 1.99) | 1.71 (1.65, 1.79) | 1,349 | 1.10 (0.98, 1.23) | 1.07 (0.96, 1.20) |
|  | Obese class II-III | 1,818 | 2.59 (2.45, 2.73) | 2.17 (2.06, 2.29) | 486 | 1.11 (0.94, 1.31) | 1.07 (0.90, 1.26) |
| **Denmark** | Underweight | 389 | 1.16 (1.04, 1.29) | 1.05 (0.94, 1.17) | 110 | 0.88 (0.68, 1.14) | 0.89 (0.69, 1.16) |
|  | Normal weight | 5367 | Reference | Reference | 589 | Reference | Reference |
|  | Overweight | 2260 | 1.28 (1.22, 1.35) | 1.25 (1.19, 1.31) | 714 | 1.02 (0.90, 1.16) | 1.02 (0.90, 1.17) |
|  | Obese class I | 992 | 1.48 (1.38, 1.59) | 1.39 (1.29, 1.49) | 385 | 1.08 (0.88, 1.33) | 1.07 (0.87, 1.31) |
|  | Obese class II-III | 661 | 1.89 (1.74, 2.06) | 1.68 (1.54, 1.84) | 150 | 0.99 (0.74, 1.31) | 0.97 (0.73, 1.29) |

Abbreviations: ASD, autism spectrum disorder; BMI, body mass index; CI, confidence interval; HR, hazard ratio.

Examination for familial confounding using a sibling-comparison design in 993,860 full siblings from 447,577 families in Sweden and 441,611 full siblings from 200,941 families in Denmark.

^a^ BMI categories were defined according to the World Health Organization as BMI <18.5, underweight; BMI 18.5-24.9, normal weight; BMI 25.0-29.9, overweight; BMI 30-34.9, obese class I; BMI ≥35, obese class II-III.

^b^ Repeating the Cox regression model I+III from the full population on the sub-population with siblings in the cohort, without stratifying on the family.

^c^ Stratified Cox regression model clustered on the mother to remove confounding from factors shared within families.

^d^ Adjusted for birth year.

^e^ Adjusted for birth year, parental age, parental educational level, disposable income, and psychiatric history

^f^ Events are informative in stratified Cox regression if the index person diagnosed with ASD have at least one discordantly exposed sibling who is still ‘at risk’, i.e. alive and not yet diagnosed with ASD at the same age as the index person.

**Table S4** Maternal BMI category and relative risk of offspring ASD in subgroups by gestational week in Sweden^a^

| **Gestational week** | **No. of children (%)** | **Underweight**  **HR (95% CI)^b^** | **Overweight**  **HR (95% CI)^b^** | **Obese class I**  **HR (95% CI)^b^** | **Obese class II-III**  **HR (95% CI)^b^** |
| --- | --- | --- | --- | --- | --- |
| <35 | 36,748 (2.6) | 1.00 (0.75, 1.35) | 1.28 (1.14, 1.43) | 1.51 (1.30, 1.75) | 1.91 (1.60, 2.27) |
| 35-36 | 27,461 (1.9) | 1.16 (0.79, 1.69) | 1.21 (1.05, 1.40) | 1.53 (1.26, 1.85) | 1.68 (1.32, 2.14) |
| 37-38 | 255,459 (17.8) | 1.26 (1.11, 1.44) | 1.27 (1.21, 1.34) | 1.61 (1.50, 1.73) | 2.09 (1.92, 2.28) |
| 39-41 | 1,008,436 (70.4) | 1.13 (1.04, 1.23) | 1.27 (1.24, 1.31) | 1.66 (1.60, 1.73) | 2.07 (1.96, 2.18) |
| >41 | 104,392 (7.3) | 1.33 (1.00, 1.78) | 1.24 (1.14, 1.34) | 1.60 (1.44, 1.79) | 2.17 (1.91, 2.47) |

Abbreviations: ASD, autism spectrum disorder; BMI, body mass index; CI, confidence interval; HR, hazard ratio.

^a^ BMI categories were defined according to the World Health Organization as BMI <18.5, underweight; BMI 18.5-24.9, normal weight; BMI 25.0-29.9, overweight; BMI 30-34.9, obese class I; BMI ≥35, obese class II-III. 103 children who were missing data on gestational age were excluded from the analysis.

^b^ Cox regression model with attainted age of child as underlying time scale and normal weight as reference, adjusted for birth year, parental age, parental educational level, disposable income, and psychiatric history.

**Table S5** Mediation of the association between maternal obesity (BMI ≥30) and offspring ASD by preterm and early-term birth (<39 weeks' gestation) in Sweden^a^

| **Model** | **Total effect** | **Natural direct effect**  **OR (95%CI)** | **Natural indirect effect OR (95%CI)** | **Percentage mediated (95% CI)** |
| --- | --- | --- | --- | --- |
| Model 1^b^ | 1.88 (1.83, 1.93) | 1.87 (1.82, 1.92) | 1.01 (1.01, 1.01) | 1.5 (1.3, 1.7) |
| Model 3^c^ | 1.66 (1.62, 1.70) | 1.65 (1.61, 1.70) | 1.00 (1.00, 1.00) | 1.0 (0.8, 1.2) |

Abbreviations: ASD, autism spectrum disorder; BMI, body mass index; CI, confidence interval; OR, odds ratio.

^a^ Mediation analysis with preterm and early-term birth as the mediator were performed by fitting natural effects models using the CAUSALMED procedure in the SAS software. BMI was dichotomized at BMI ≥30 (obese) versus <30 (not obese). Analyses consist of two logistic regression models; an outcome model including obesity as a predictor of offspring ASD risk and a mediator model including obesity as a predictor of preterm or early-term birth. The number of children born preterm or early term was 42,878 (25.0%) among mothers with BMI ≥30 and 276,790 (22.0%) among mother with BMI <30. 103 children who were missing data on gestational age were excluded from the analysis.

^b^ Adjusted for birth year.

^c^ Adjusted for birth year, parental age, educational level, disposable income, and psychiatric history.

**Table S6** Maternal BMI category and relative risk of offspring ASD for birth years 2004-2018 in Sweden with follow-up until 31 December 2021

| **BMI category^a^** | **Rate (cases/ 100,000 person years)** | **ASD cases/person-years** | **Model 1^b^  HR (95% CI)** | **Model 2^c^  HR (95% CI)** | **Model 3^d^ HR (95% CI)** |
| --- | --- | --- | --- | --- | --- |
| Underweight | 288.9 | 484/167,531 | 1.37 (1.25, 1.51) | 1.18 (1.07, 1.29) | 1.15 (1.04, 1.26) |
| Normal weight | 213.5 | 11,156/5,225,384 | Reference | Reference | Reference |
| Overweight | 286.4 | 6,090/2,126,563 | 1.35 (1.31, 1.39) | 1.29 (1.24, 1.33) | 1.29 (1.25, 1.33) |
| Obese class I | 395.2 | 2,786/705,004 | 1.88 (1.81, 1.97) | 1.67 (1.60, 1.75) | 1.67 (1.60, 1.75) |
| Obese class II-III | 546.6 | 1,697/310,437 | 2.63 (2.49, 2.77) | 2.20 (2.08, 2.32) | 2.18 (2.07, 2.31) |

Abbreviations: ASD, autism spectrum disorder; BMI, body mass index; CI, confidence interval; HR, hazard ratio.

^a^ BMI categories were defined according to the World Health Organization as BMI <18.5, underweight; BMI 18.5-24.9, normal weight; BMI 25.0-29.9, overweight; BMI 30-34.9, obese class I; BMI ≥35, obese class II-III.

^b^ Cox regression model with attainted age of child as underlying time scale, adjusted for birth year.

^c^ Adjusted for birth year, parental age, educational level, and disposable income.

^d^ Adjusted for birth year, parental age, educational level, disposable income, and psychiatric history.

**Table S7** Maternal BMI category and relative risk of offspring ASD from multiply imputed data in Sweden

| **BMI categories^a^** | **Model 1^b^  HR (95% CI)** | **Model 2^c^  HR (95% CI)** | **Model 3^d^ HR (95% CI)** |
| --- | --- | --- | --- |
| Underweight | 1.46 (1.37, 1.55) | 1.25 (1.17, 1.33) | 1.22 (1.15, 1.30) |
| Normal weight | Reference | Reference | Reference |
| Overweight | 1.34 (1.31, 1.37) | 1.27 (1.25, 1.30) | 1.28 (1.25, 1.31) |
| Obese class I | 1.83 (1.78, 1.88) | 1.64 (1.59, 1.69) | 1.63 (1.58, 1.68) |
| Obese class II-III | 2.43 (2.34, 2.52) | 2.06 (1.98, 2.14) | 2.04 (1.96, 2.12) |

Abbreviations: ASD, autism spectrum disorder; BMI, body mass index; CI, confidence interval; HR, hazard ratio.

^a^ BMI categories were defined according to the World Health Organization as BMI <18.5, underweight; BMI 18.5-24.9, normal weight; BMI 25.0-29.9, overweight; BMI 30-34.9, obese class I; BMI ≥35, obese class II-III.

^b^ Cox regression model with attainted age of child as underlying time scale, adjusted for birth year.

^c^ Adjusted for birth year, parental age, educational level, and disposable income.

^d^ Adjusted for birth year, parental age, educational level, disposable income, and psychiatric history.

**Table S8** Cohort characteristics by maternal BMI category including subjects with missing data in Sweden^a^

|  | **Maternal BMI category in early pregnancy** | | | | | |
| --- | --- | --- | --- | --- | --- | --- |
| **Characteristic** | **Underweight** | **Normal weight** | **Overweight** | **Obese class I** | **Obese class II-III** | **BMI missing** |
| Number of children (% of total) | 29,247 (1.8) | 878,577 (55.3) | 361,381 (22.8) | 120,477 (7.6) | 52,810 (3.3) | 145,162 (9.1) |
| Male sex | 14,706 (50.3) | 452,360 (51.5) | 185,838 (51.4) | 62,193 (51.6) | 27,265 (51.6) | 75,084 (51.7) |
| Birth year |  |  |  |  |  |  |
| 1998-2001 | 4,822 (16.5) | 143,636 (16.3) | 56,811 (15.7) | 16,479 (13.7) | 6,068 (11.5) | 38,528 (26.5) |
| 2002-2004 | 3,583 (12.3) | 118,880 (13.5) | 47,834 (13.2) | 14,713 (12.2) | 6,230 (11.8) | 27,301 (18.8) |
| 2005-2007 | 3,837 (13.1) | 122,520 (13.9) | 49,451 (13.7) | 15,797 (13.1) | 7,028 (13.3) | 25,420 (17.5) |
| 2008-2010 | 4,218 (14.4) | 131,286 (14.9) | 52,815 (14.6) | 17,546 (14.6) | 7,849 (14.9) | 15,082 (10.4) |
| 2011-2013 | 4,323 (14.8) | 126,135 (14.4) | 52,034 (14.4) | 18,024 (15.0) | 7,843 (14.9) | 13,546 (9.3) |
| 2014-2016 | 4,496 (15.4) | 123,818 (14.1) | 51,000 (14.1) | 18,518 (15.4) | 8,392 (15.9) | 12,787 (8.8) |
| 2017-2019 | 3,968 (13.6) | 112,302 (12.8) | 51,436 (14.2) | 19,400 (16.1) | 9,400 (17.8) | 12,498 (8.6) |
| Maternal age, years |  |  |  |  |  |  |
| <25 | 7,029 (24.0) | 104,107 (11.8) | 44,633 (12.4) | 17,529 (14.5) | 7,866 (14.9) | 17,985 (12.4) |
| 25-34 | 18,575 (63.5) | 603,571 (68.7) | 238,879 (66.1) | 77,742 (64.5) | 34,092 (64.6) | 95,623 (65.9) |
| ≥35 | 3,643 (12.5) | 170,894 (19.5) | 77,867 (21.5) | 25,205 (20.9) | 10,851 (20.5) | 31,553 (21.7) |
| Missing | 0 (<0.1) | 5 (<0.1) | 2 (<0.1) | 1 (<0.1) | 1 (<0.1) | 1 (<0.1) |
| Paternal age, years |  |  |  |  |  |  |
| <25 | 3,887 (13.3) | 53,802 (6.1) | 22,455 (6.2) | 8,489 (7.0) | 3,582 (6.8) | 9,549 (6.6) |
| 25-34 | 17,693 (60.5) | 530,553 (60.4) | 211,921 (58.6) | 69,199 (57.4) | 29,556 (56.0) | 83,943 (57.8) |
| 35-42 | 6,552 (22.4) | 252,711 (28.8) | 106,886 (29.6) | 35,539 (29.5) | 16,013 (30.3) | 43,188 (29.8) |
| ≥43 | 1,115 (3.8) | 41,511 (4.7) | 20,119 (5.6) | 7,250 (6.0) | 3,659 (6.9) | 8,482 (5.8) |
| **Characteristic** | **Underweight** | **Normal weight** | **Overweight** | **Obese class I** | **Obese class II-III** | **BMI missing** |
| Maternal psychiatric history | 4,951 (16.9) | 97,484 (11.1) | 44,007 (12.2) | 17,682 (14.7) | 8,870 (16.8) | 15,270 (10.5) |
| Paternal psychiatric history | 3,094 (10.6) | 62,658 (7.1) | 29,868 (8.3) | 12,672 (10.5) | 6,678 (12.6) | 10,067 (6.9) |
| Maternal education |  |  |  |  |  |  |
| Compulsory | 3,914 (13.4) | 55,221 (6.3) | 27,830 (7.7) | 12,340 (10.2) | 6,544 (12.4) | 12,653 (8.7) |
| Upper secondary | 12,169 (41.6) | 337,306 (38.4) | 169,763 (47.0) | 64,972 (53.9) | 30,600 (57.9) | 61,099 (42.1) |
| University | 12,957 (44.3) | 483,676 (55.1) | 162,856 (45.1) | 42,760 (35.5) | 15,429 (29.2) | 70,774 (48.8) |
| Missing | 207 (0.7) | 2,374 (0.3) | 932 (0.3) | 405 (0.3) | 237 (0.4) | 636 (0.4) |
| Paternal education |  |  |  |  |  |  |
| Compulsory | 3,836 (13.1) | 73,731 (8.4) | 37,056 (10.3) | 15,428 (12.8) | 7,659 (14.5) | 14,797 (10.2) |
| Upper secondary | 14,110 (48.2) | 416,375 (47.4) | 204,892 (56.7) | 75,328 (62.5) | 34,342 (65.0) | 71,724 (49.4) |
| University | 11,153 (38.1) | 385,662 (43.9) | 118,320 (32.7) | 29,240 (24.3) | 10,539 (20.0) | 57,996 (40.0) |
| Missing | 148 (0.5) | 2,809 (0.3) | 1,113 (0.3) | 481 (0.4) | 270 (0.5) | 645 (0.4) |
| Maternal disposable income^b^ |  |  |  |  |  |  |
| Q1 | 10,989 (37.6) | 204,616 (23.3) | 90,102 (24.9) | 35,748 (29.7) | 18,300 (34.7) | 36,785 (25.3) |
| Q2 | 6,828 (23.3) | 208,350 (23.7) | 96,738 (26.8) | 35,516 (29.5) | 15,913 (30.1) | 33,244 (22.9) |
| Q3 | 5,551 (19.0) | 221,168 (25.2) | 94,576 (26.2) | 29,316 (24.3) | 11,899 (22.5) | 34,021 (23.4) |
| Q4 | 5,833 (19.9) | 243,678 (27.7) | 79,700 (22.1) | 19,817 (16.4) | 6,681 (12.7) | 40,844 (28.1) |
| Missing | 46 (0.2) | 765 (0.1) | 265 (0.1) | 80 (0.1) | 17 (<0.1) | 268 (0.2) |
| Paternal disposable income^b^ |  |  |  |  |  |  |
| Q1 | 9,750 (33.3) | 207,813 (23.7) | 89,742 (24.8) | 34,447 (28.6) | 17,545 (33.2) | 36,992 (25.5) |
| Q2 | 6,479 (22.2) | 205,811 (23.4) | 98,960 (27.4) | 36,146 (30.0) | 16,166 (30.6) | 32,898 (22.7) |
| Q3 | 6,101 (20.9) | 217,177 (24.7) | 95,379 (26.4) | 30,842 (25.6) | 12,754 (24.2) | 34,196 (23.6) |
| Q4 | 6,866 (23.5) | 246,598 (28.1) | 76,945 (21.3) | 18,937 (15.7) | 6,305 (11.9) | 40,736 (28.1) |
| Missing | 51 (0.2) | 1,178 (0.1) | 355 (0.1) | 105 (0.1) | 40 (0.1) | 340 (0.2) |

Abbreviations: BMI, body mass index.

^a^ Data are expressed as number (percentage) unless otherwise indicated. BMI categories were defined according to the World Health Organization as BMI <18.5, underweight; BMI 18.5-24.9, normal weight; BMI 25.0-29.9, overweight; BMI 30-34.9, obese class I; BMI ≥35, obese class II-III.

^b^ In quartiles per income year.

**SUPPLEMENTARY FIGURES:**

**Figure S1** Flow diagram over study cohort selection

**
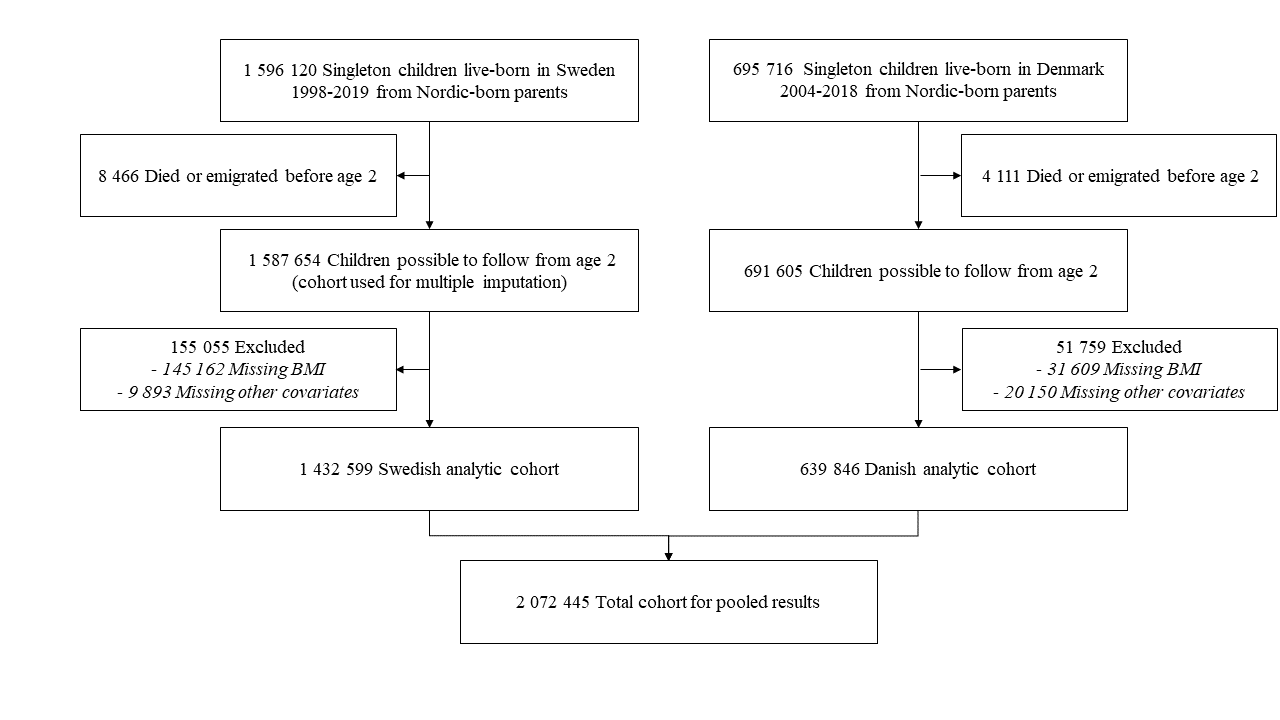
**

**Figure S2** Scaled Schoenfeld residual plots to assess proportional hazards assumption (Swedish cohort)


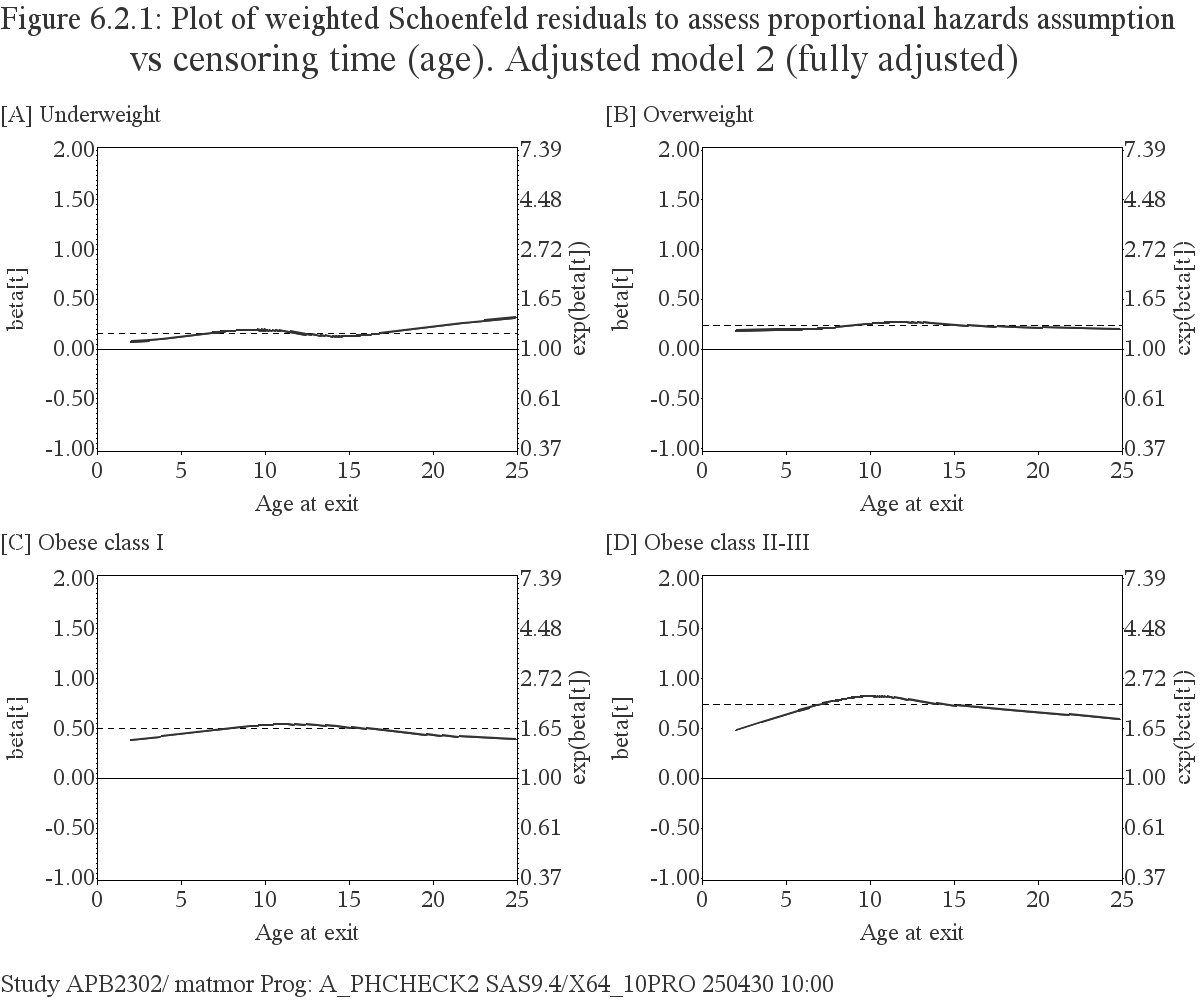


Model 3 adjusted for birth year, parental age, educational level, disposable income, and psychiatric history. Left vertical axis is on log scale and right vertical axis on anti-log scale. Horizontal dashed line correspond to mean residual which approximate the Cox regression hazard ratio for the same parameter. BMI categories were defined according to the World Health Organization as BMI <18.5, underweight; BMI 18.5-24.9, normal weight; BMI 25.0-29.9, overweight; BMI 30-34.9, obese class I; BMI ≥35, obese class II-III.

**Figure S3** Scaled Schoenfeld residual plots to assess proportional hazards assumption (Danish cohort)


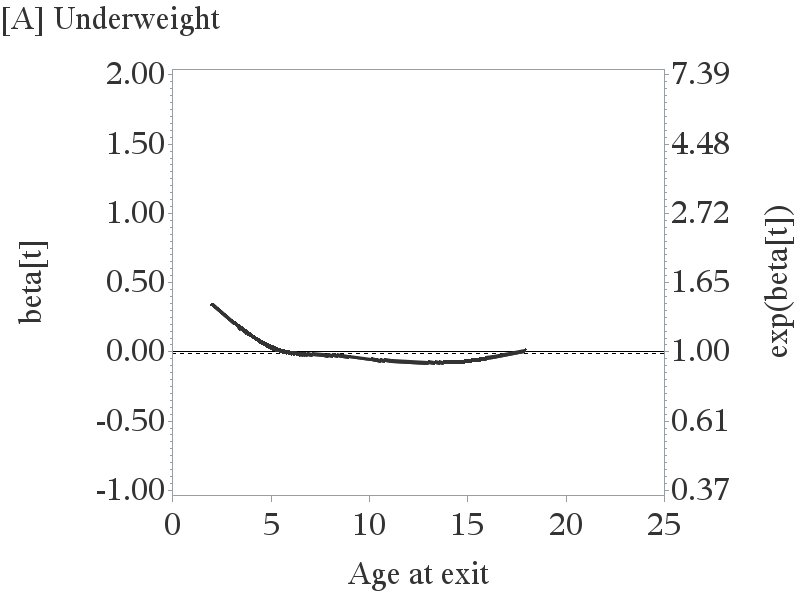

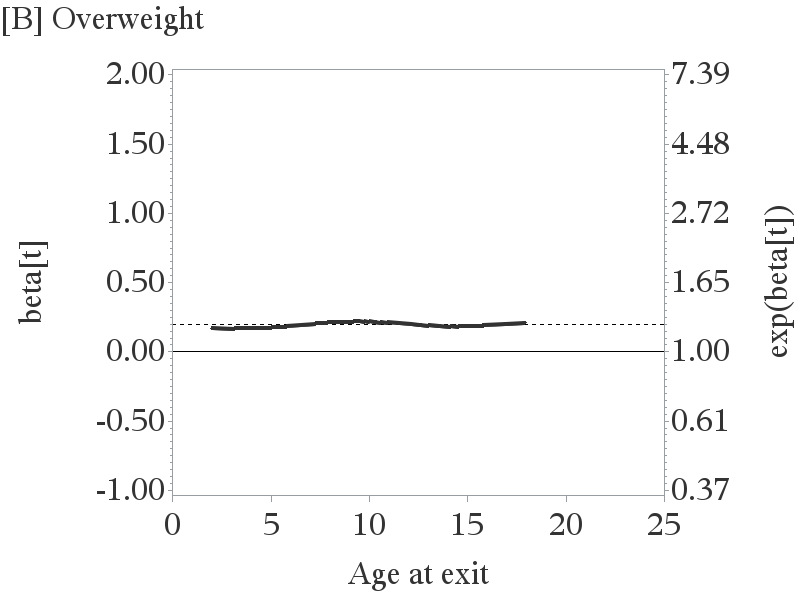

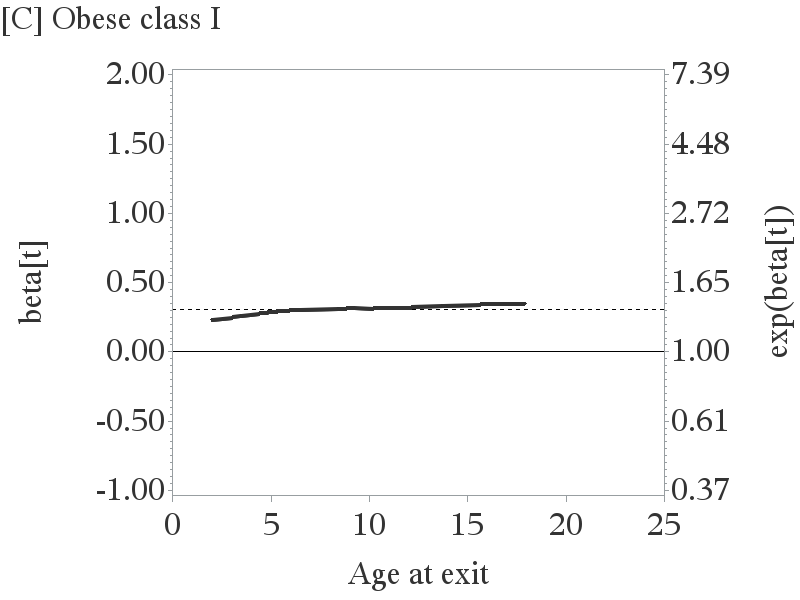

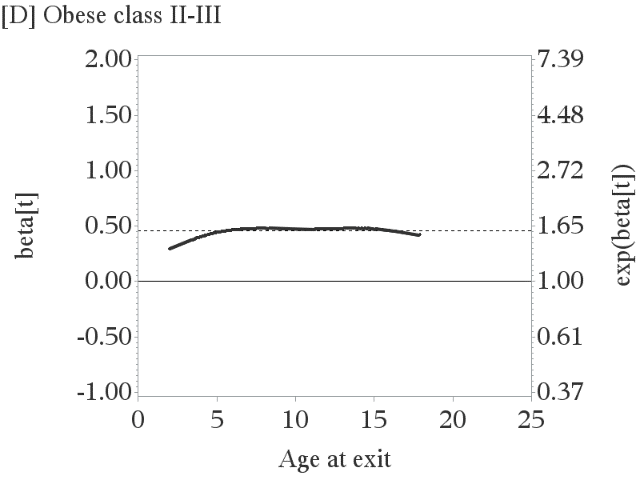


Model 3 adjusted for birth year, parental age, educational level, disposable income, and psychiatric history. Left vertical axis is on log scale and right vertical axis on anti-log scale. Horizontal dashed line correspond to mean residual which approximate the Cox regression hazard ratio for the same parameter. BMI categories were defined according to the World Health Organization as BMI <18.5, underweight; BMI 18.5-24.9, normal weight; BMI 25.0-29.9, overweight; BMI 30-34.9, obese class I; BMI ≥35, obese class II-III.

**Figure S4** Maternal body mass index (BMI) (continuous) and relative risk of offspring autism spectrum disorder in (A) Sweden and (B) Denmark


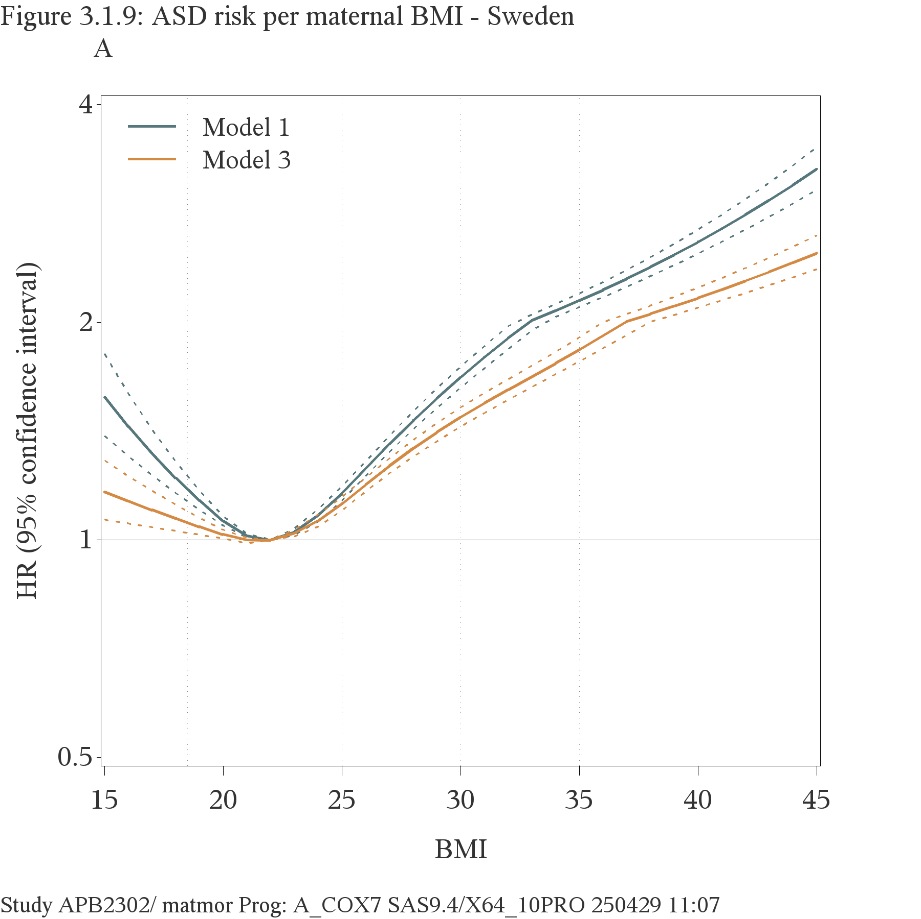

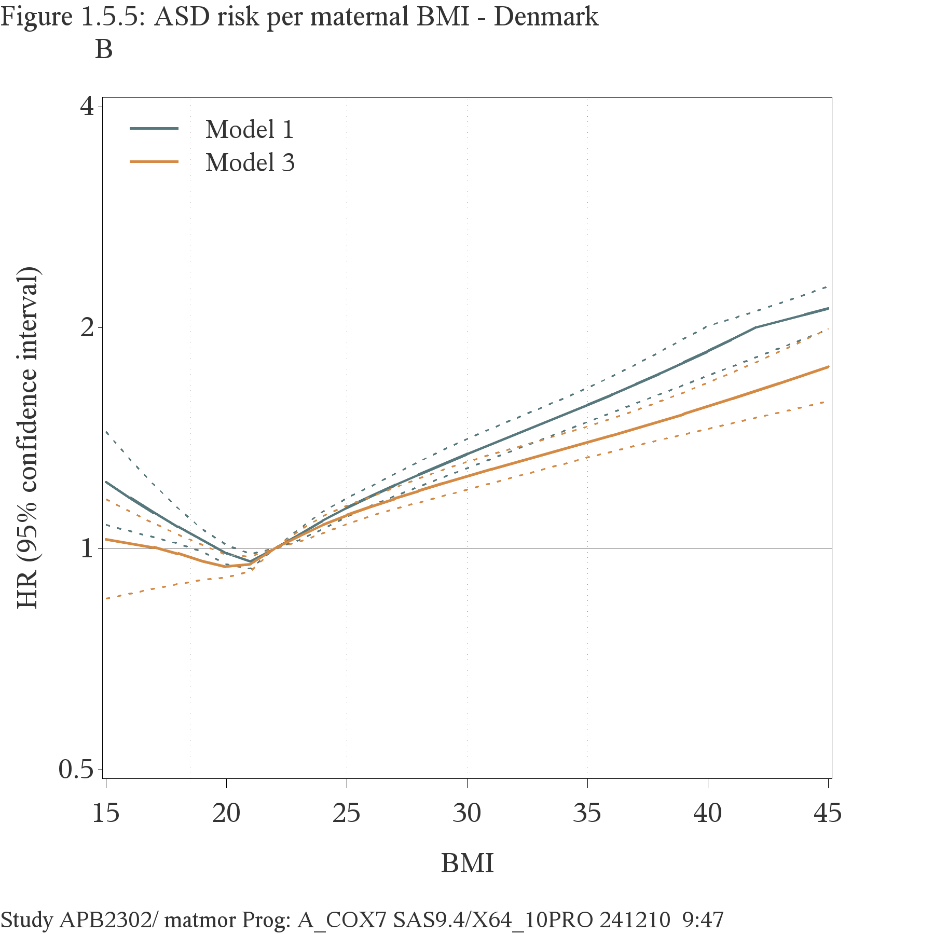


Hazard ratios (HRs) from Cox regression models with attainted age of child as underlying time scale, adjusted for birth year (model 1), plus parental age, educational level, disposable income, and psychiatric history (model 3). BMI was modeled with a restricted cubic spline, using the median BMI value in the ‘normal weight’ category, i.e. 22, as reference. Dashed lines indicated 95% confidence intervals.

**Figure S5** Maternal body mass index (BMI) category and relative risk of offspring autism spectrum disorder (ASD) with co-occurring intellectual disability (ID) or attention deficit hyperactivity disorder (ADHD) in Sweden and Denmark


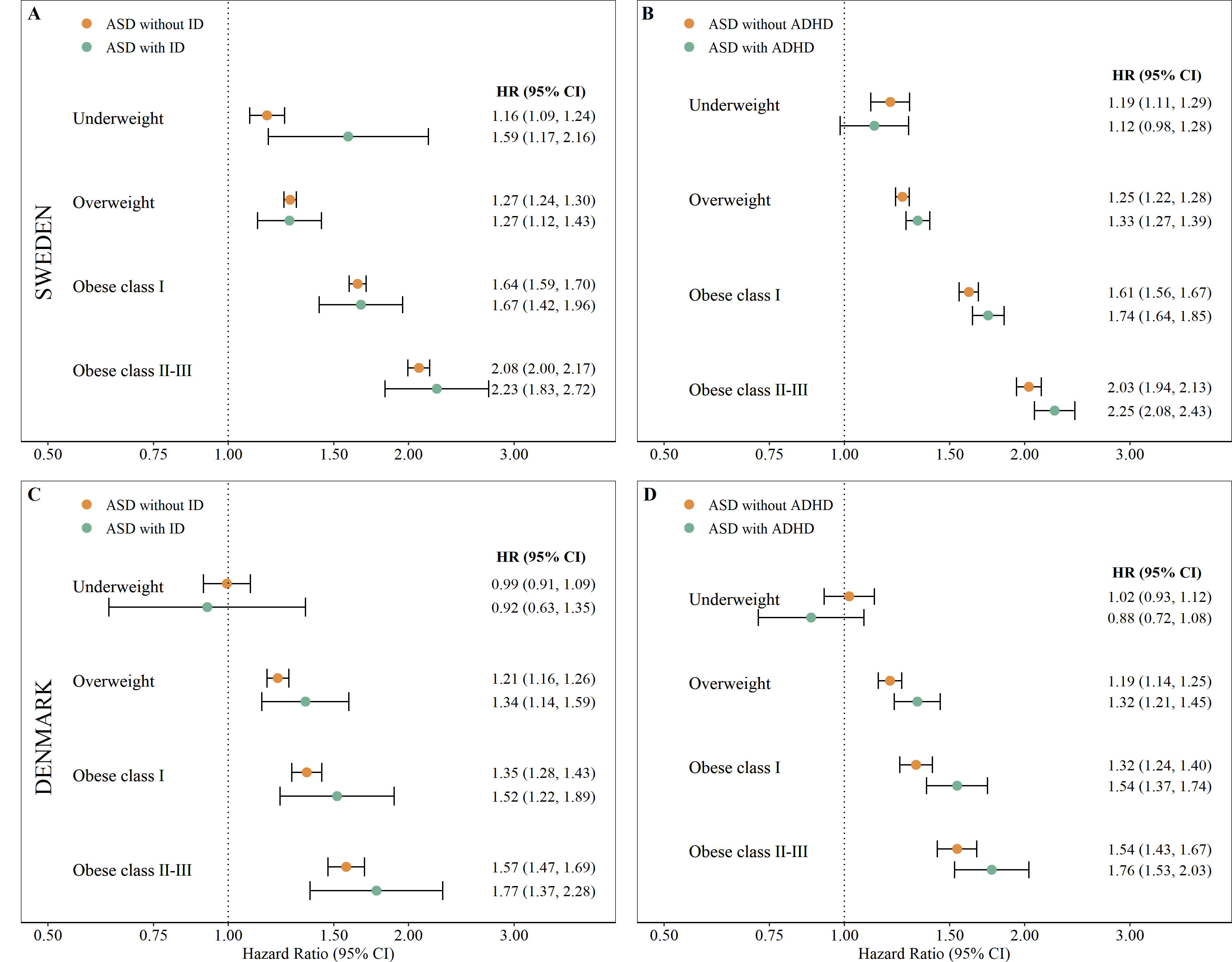


Dots represent hazard ratios (HRs) with 95% confidence interval bars for ASD with or without co-occurring ID (panel A+C) or ADHD (panel B+D) by maternal BMI category in Sweden (panel A+B) and Denmark (panel C+D). Results from Cox regression models adjusted for birth year, parental age, educational level, disposable income, and psychiatric history (model 3). BMI categories were defined according to the World Health Organization as BMI <18.5, underweight; BMI 18.5-24.9, normal weight; BMI 25.0-29.9, overweight; BMI 30-34.9, obese class I; BMI ≥35, obese class II-III. **Figure S6** Maternal body mass index (BMI) (continuous) and relative risk of offspring autism spectrum disorder in full siblings in (A) Sweden and (B) Denmark


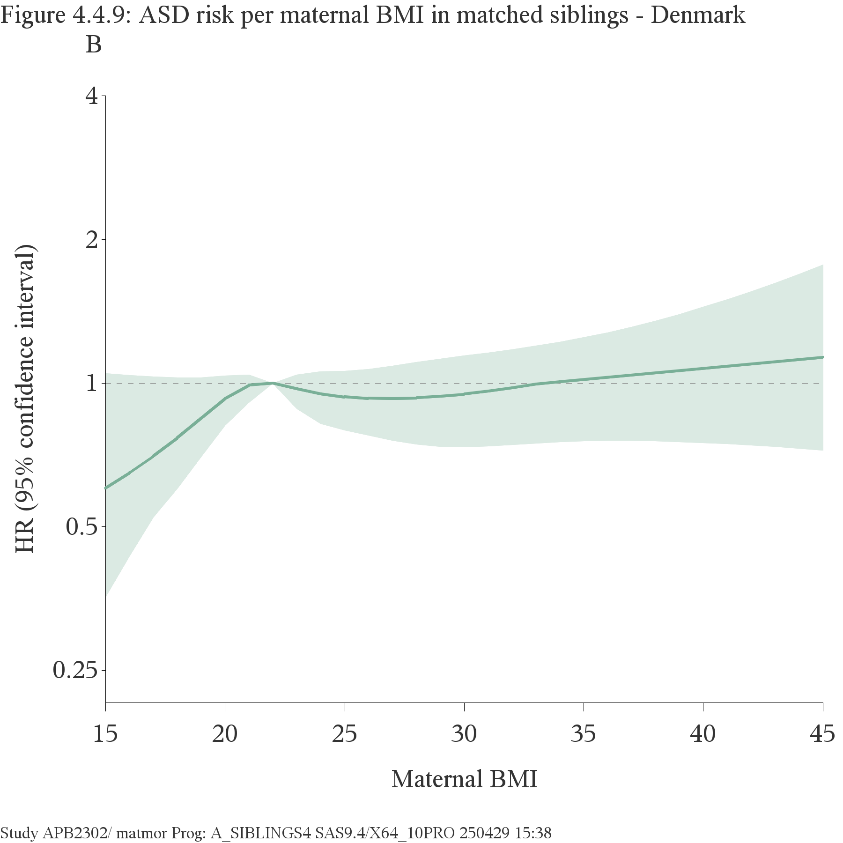

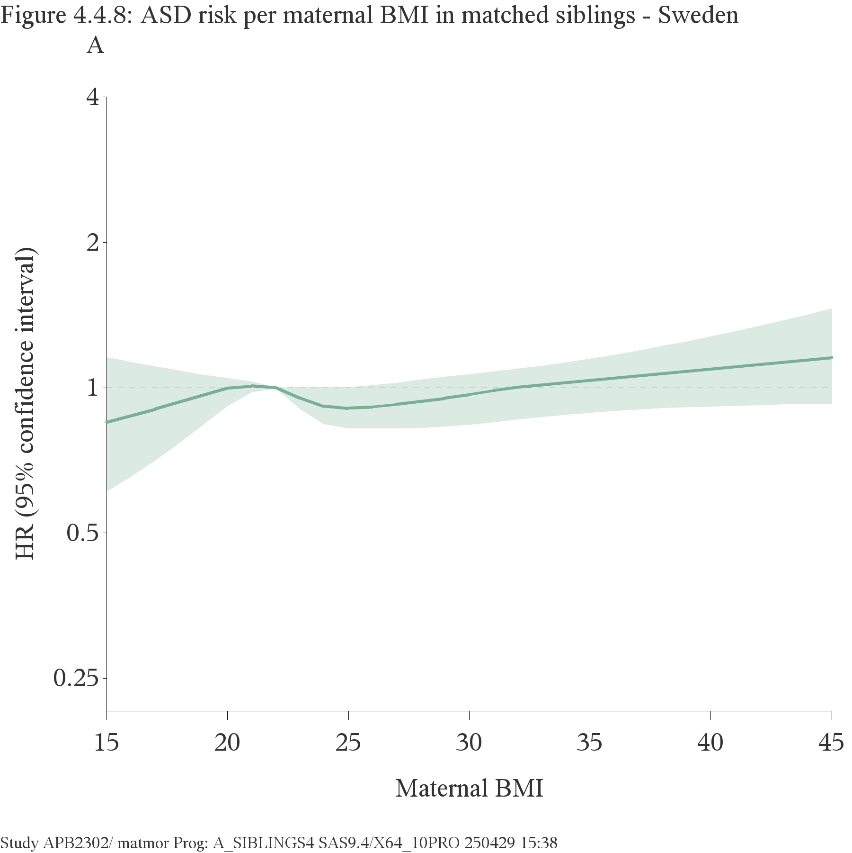


Hazard ratios (HRs) from stratified Cox regression models adjusted for birth year, parental age, educational level, disposable income, and psychiatric history (model 3). BMI was modeled with a restricted cubic spline, using the median BMI value in the ‘normal weight’ category, i.e. 22, as reference. Shaded bands indicate 95% confidence intervals.

**Figure S7** Maternal body mass index (BMI) (continuous) and relative risk of offspring autism spectrum disorder by offspring sex in (A) Sweden and (B) Denmark


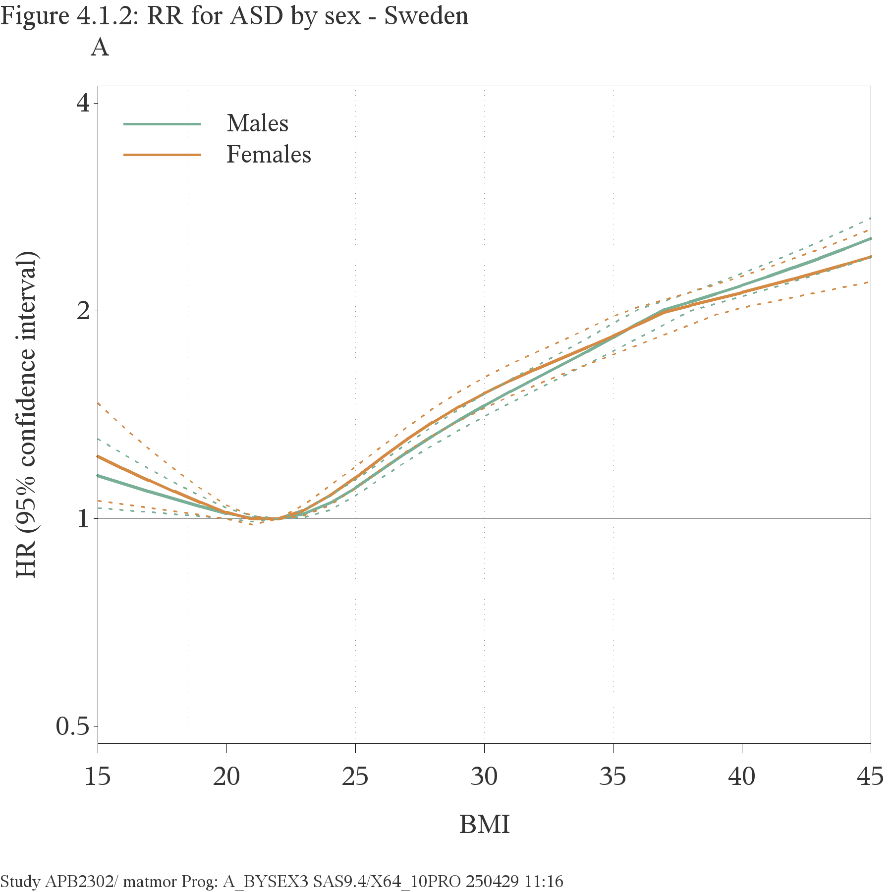

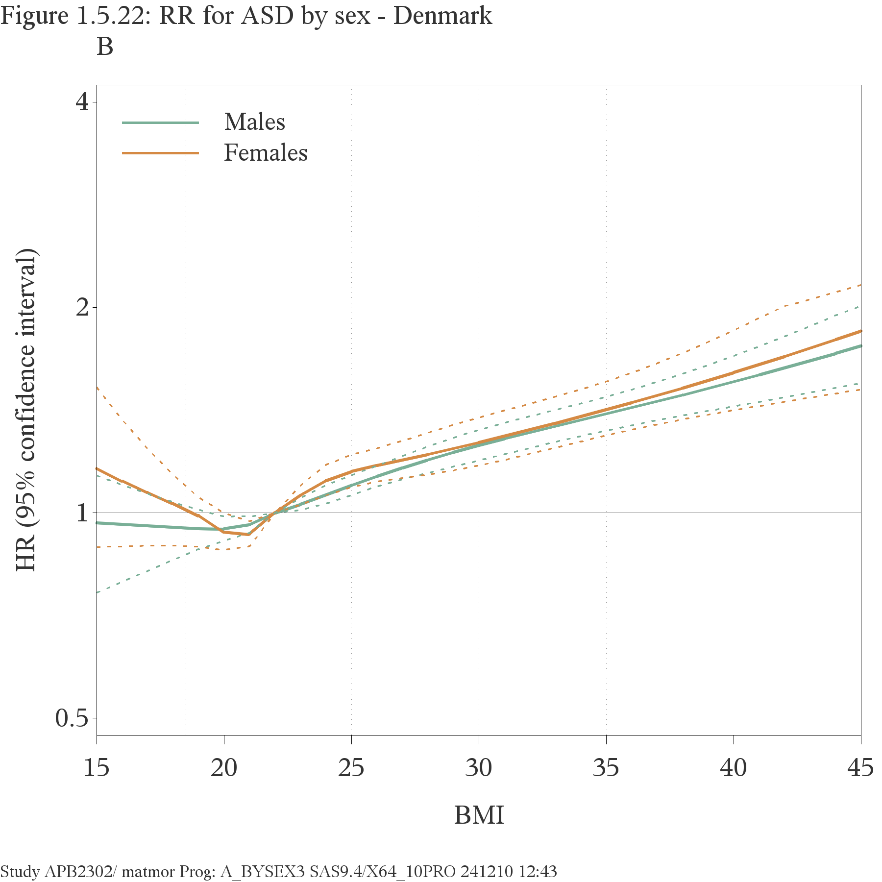


Hazard ratios (HRs) from Cox regression model stratified by sex, with attainted age of child as underlying time scale, adjusted for birth year, parental age, educational level, disposable income and psychiatric history (model 3). BMI was modeled with a restricted cubic spline, using the median BMI value in the ‘normal weight’ category, i.e. 22, as reference. Dashed lines indicated 95% confidence intervals.

**Figure S8** Maternal body mass index (BMI) category and pooled relative risk of offspring autism spectrum disorder (ASD) with co-occurring (A) intellectual disability (ID), or (B) attention deficit hyperactivity disorder (ADHD)


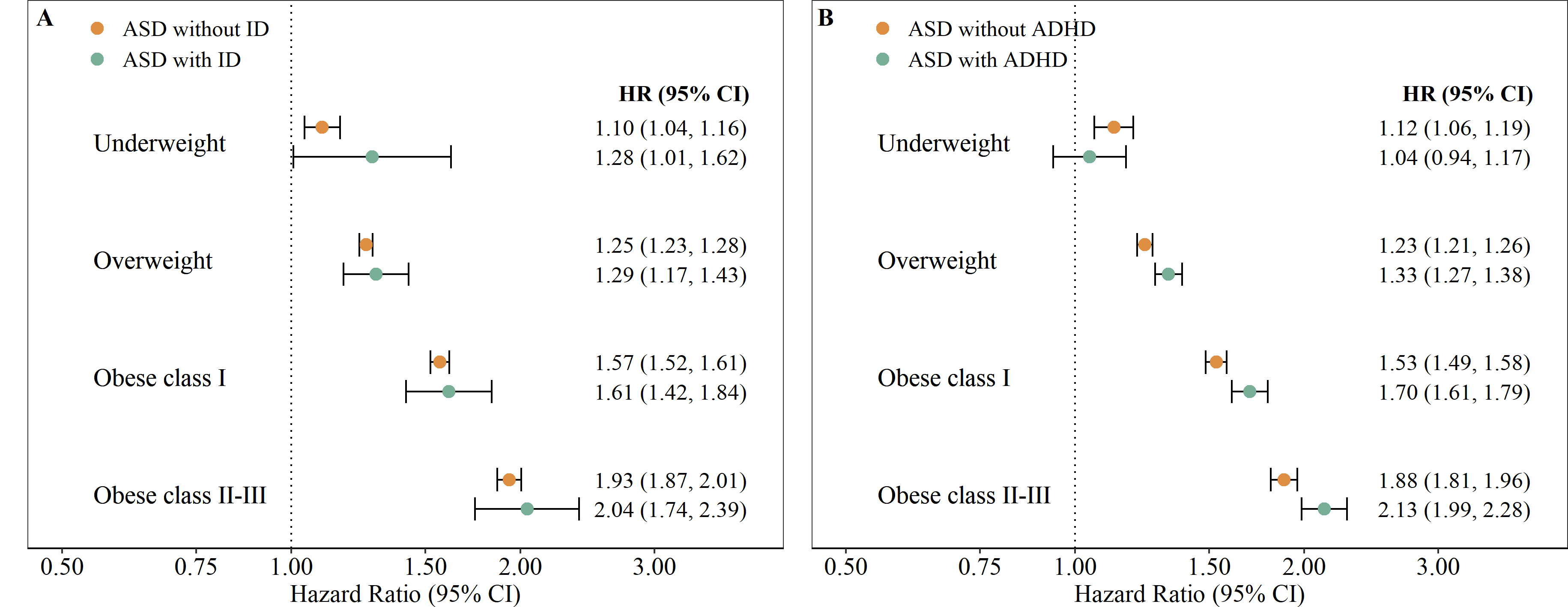


Dots represent hazard ratios (HRs) with 95% confidence interval bars from Cox regression models adjusted for birth year, parental age, educational level, disposable income, and psychiatric history (model 3). BMI categories were defined according to the World Health Organization as BMI <18.5, underweight; BMI 18.5-24.9, normal weight; BMI 25.0-29.9, overweight; BMI 30-34.9, obese class I; BMI ≥35, obese class II-III.

**Figure S9** Predicted probability of offspring autism spectrum disorder in relation to maternal body mass index (BMI) difference in a specific pregnancy from the mother’s median BMI over all her pregnancies, by categories of median maternal BMI (Swedish cohort)


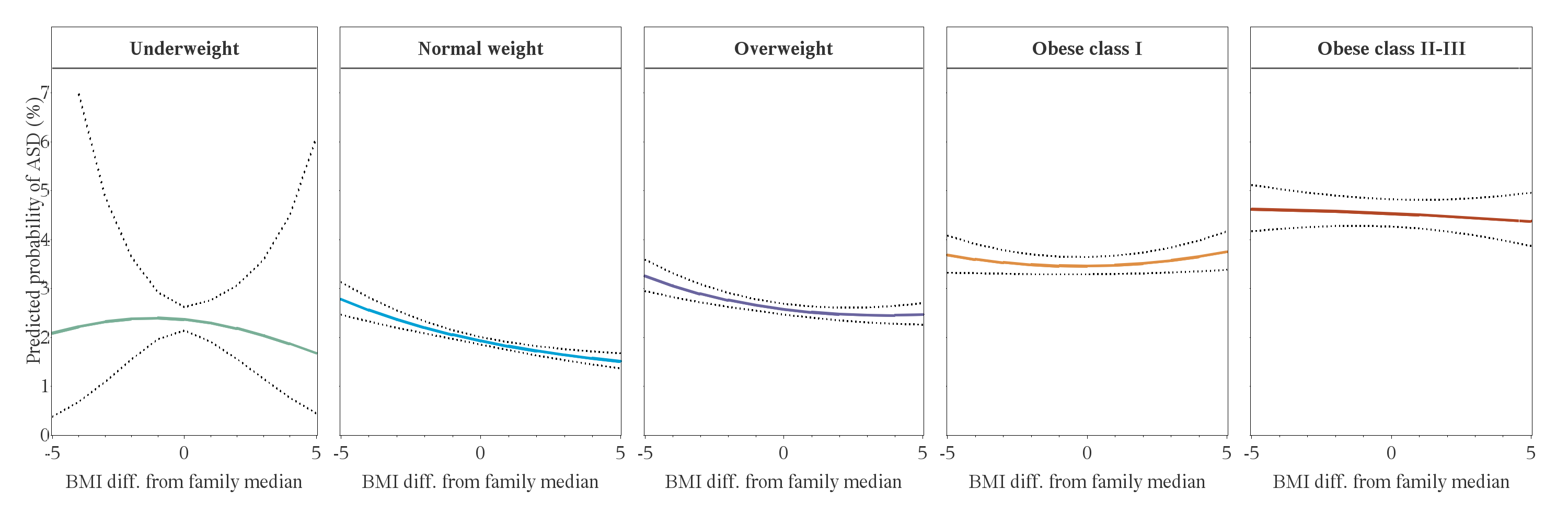


For each mother with at least 2 children in the cohort, the median BMI over all her pregnancies was estimated. Mothers were then classified into categories by their median BMI. The difference of maternal BMI in an individual pregnancy compared to the median was estimated for each child (referred to as BMI diff). A logistic regression was used to predict the ASD risk associated with BMI diff in the different median BMI categories. Analysis was restricted to the first 4 births of each woman. BMI diff was modelled with a second-degree polynomial. ASD risk was predicted at the median level of all covariates, i.e. birth years 2008-2010, maternal and paternal age 30-39 (categorized into 10-year groups for this analysis), maternal university education, paternal secondary school education, parental income in Q3, maternal income Q2, with no parental psychiatric history. The predicted probability of ASD was then plotted against BMI diff in each median BMI category.
